# Ancestral morphology of Ecdysozoa constrained by an early Cambrian stem group ecdysozoan

**DOI:** 10.1186/s12862-020-01720-6

**Published:** 2020-11-23

**Authors:** Richard J. Howard, Gregory D. Edgecombe, Xiaomei Shi, Xianguang Hou, Xiaoya Ma

**Affiliations:** 1 MEC International Joint Laboratory for Palaeobiology and Palaeoenvironment, Yunnan University, Chenggong Campus, Kunming, 650500 China; 2 Yunnan Key Laboratory for Palaeobiology, Institute of Palaeontology, Yunnan University, Chenggong Campus, Kunming, 650500 China; 3 Centre for Ecology and Conservation, University of Exeter, Penryn Campus, Cornwall, TR10 9TA UK; 4 Department of Earth Sciences, The Natural History Museum, Cromwell Road, London, SW7 5BD UK

**Keywords:** Ecdysozoa, Cambrian, Cycloneuralia, Panarthropoda, Palaeobiology, Phylogenetics

## Abstract

**Background:**

Ecdysozoa are the moulting protostomes, including arthropods, tardigrades, and nematodes. Both the molecular and fossil records indicate that Ecdysozoa is an ancient group originating in the terminal Proterozoic, and exceptional fossil biotas show their dominance and diversity at the beginning of the Phanerozoic. However, the nature of the ecdysozoan common ancestor has been difficult to ascertain due to the extreme morphological diversity of extant Ecdysozoa, and the lack of early diverging taxa in ancient fossil biotas.

**Results:**

Here we re-describe *Acosmia maotiania* from the early Cambrian Chengjiang Biota of Yunnan Province, China and assign it to stem group Ecdysozoa. *Acosmia* features a two-part body, with an anterior proboscis bearing a terminal mouth and muscular pharynx, and a posterior annulated trunk with a through gut. Morphological phylogenetic analyses of the protostomes using parsimony, maximum likelihood and Bayesian inference, with coding informed by published experimental decay studies, each placed *Acosmia* as sister taxon to Cycloneuralia + Panarthropoda—i.e. stem group Ecdysozoa. Ancestral state probabilities were calculated for key ecdysozoan nodes, in order to test characters inferred from fossils to be ancestral for Ecdysozoa. Results support an ancestor of crown group ecdysozoans sharing an annulated vermiform body with a terminal mouth like *Acosmia*, but also possessing the pharyngeal armature and circumoral structures characteristic of Cambrian cycloneuralians and lobopodians.

**Conclusions:**

*Acosmia* is the first taxon placed in the ecdysozoan stem group and provides a constraint to test hypotheses on the early evolution of Ecdysozoa. Our study suggests acquisition of pharyngeal armature, and therefore a change in feeding strategy (e.g. predation), may have characterised the origin and radiation of crown group ecdysozoans from *Acosmia*-like ancestors.

## Background

Ecdysozoa are the moulting invertebrates, including arthropods, tardigrades and nematodes [1, 2]. Along with the Spiralia (e.g. molluscs, flatworms and annelids) and the Deuterostomia (e.g. chordates and echinoderms), the Ecdysozoa represent one of the major subdivisions of bilaterian animals. Ecdysozoa comprises the vast majority of this bilateral animal diversity (and indeed animals generally)—principally through the megadiverse arthropods. Together with Spiralia, the ecdysozoans comprise the Protostomia. Molecular clocks indicate the divergence between Ecdysozoa and Spiralia occurred in the Ediacaran Period [3, 4], but the group does not appear in the fossil record with certainty until the base of the Cambrian [5, 6]—though some late Ediacaran trace fossils are potentially attributable to ecdysozoans [7–9]. Both cycloneuralians (worm-like ecdysozoans) and panarthropods (paired appendage-bearing ecdysozoans) then appear rapidly, marking significant stratigraphic boundaries [5, 6, 10, 11] and seemingly tracking the duration of the Cambrian Explosion itself [12]. Hypotheses concerning the origins and early evolution of multiple ecdysozoan subgroups have been proposed from their spectacular Cambrian fossil record [13–18], but all taxa fall within the Cycloneuralia (Scalidophora + Nematoida) or Panarthropoda, with little known about the ancestral characteristics of Ecdysozoa beyond character optimisation from trees of crown group taxa [14, 19]. This renders the little-known early Cambrian Chengjiang Biota taxon *Acosmia maotiania* Chen and Zhou, 1997 [20] particularly intriguing, as it possesses several widely distributed ecdysozoan characteristics (e.g. vermiform bodyplan, annulated cuticle, a terminal mouth in the presumed adult form)—but none of the particular characters diagnostic of the subgroups Panarthropoda, Nematoida or Scalidophora. Here we present a study re-describing *Acosmia maotiania*, and placing it in the ecdysozoan stem-lineage through phylogenetic analysis.

*Acosmia* has been reported as a burrowing, deposit-feeding priapulan, based on its “U”-shaped fossils and infilled through gut [20]—suggesting perhaps a lugworm-like lifestyle. The animal does somewhat resemble a megaintrovertan priapulan (e.g. *Priapulus* sp.) in general shape, with an annulated cuticle and an expanded anterior region that takes up a relatively large portion of its total length. However, *Acosmia* appears to lack key characteristics that are diagnostic of priapulans and other scalidophorans [21], including the retractable anterior introvert and pharyngeal teeth. As such, *Acosmia* has been considered to be of uncertain classification in subsequent reviews [22–25]. The anterior region in *Acosmia* shows no sign of eversibility, and it lacks the parallel longitudinal arrangement of armature (known as “scalids”) that is characteristic of crown group priapulans, and their hypothesised stem groups the archaeopriapulids and palaeoscolecids [17]. In fact, *Acosmia* appears to lack this kind of armature altogether. Scalids are hollow and radially arranged sensory and locomotive structures that adorn the introverts of all priapulans, kinorhynchs and loriciferans [21, 26], and give rise to the clade name Scalidophora. Unsurprisingly, these diverse but regularly arranged armature structures on the proboscis region are a chief diagnostic character in recognising fossil scalidophorans. They may be preserved in high fidelity in Chengjiang scalidophorans as reddish or dark-coloured spines or compressed spots [27], and also have a rich Cambrian record as carbonaceous microfossils [28]. Decay experiments on the extant priapulan *Priapulus caudatus* show that scalids are highly recalcitrant tissues that persist long into the decay process, along with other elements of the cuticular anatomy [29]. Despite the lack of scalids in *Acosmia* material, other such recalcitrant cuticular structures are preserved*,* including distinct anterior and posterior papillae and trunk annulations. Therefore, the absence of scalids on the anterior region of *Acosmia* is unlikely to be a taphonomic artefact, and it is more likely that *Acosmia* did not possess a scalid-covered introvert. *Acosmia* also lacks the caudal appendage(s) possessed by most priapulans, including coeval priapulan fossils such as *Xiaoheiqingella* [25, 27], and shows no sign of pharyngeal eversibility. As such, *Acosmia’*s status as a priapulan is doubtful.

An updated description of *Acosmia maotiania* is provided based on examination of new and historic fossil material, with a total of seven of nine known individuals documented. Sampling widely across the protostomes, a phylogenetic matrix was compiled and scored, comprising 185 characters for 62 taxa (*Acosmia*, 25 spiralian terminals, 35 ecdysozoan terminals, and 1 deuterostome outgroup). Phylogenies were inferred from this matrix using both parsimony and probabilistic methods, all recovering *Acosmia* as a stem group ecdysozoan. Ancestral character state probabilities for key morphological characters were then calculated under alternative topological hypotheses in order to elucidate the nature of the ancestral ecdysozoan—newly constrained by the systematic position and character states of *Acosmia*.

## Results

### Systematic palaeontology

#### Superphylum

Ecdysozoa Aguinaldo et al. 1997 [1]

**Table:** 

	Genus and species	
1997	*Acosmia maotiania*	Chen and Zhou [20]
1999	*Acosmia maotiania*	Hou et al. [22]
2004	*Acosmia maotiania*	Hou et al. [23]
2017	*Acosmia maotiania*	Hou et al. [25]

#### Type material

Holotype ELRC 51001 figured in Chen & Zhou [20]. See Table 1 for complete list of referred material.

**Table 1:** Referred material

Name	Accession	Source and illustration	Comments
*Acosmia maotiania*	ELRC 51001	Figure 31 in [20]	Holotype. Part and counterpart
*Acosmia maotiania*	ELRC 51002	Figure 33 in [20]	Part and counterpart
*Acosmia maotiania*	RCCBYU 10233	Figure 12.3a in [23] Figure 1 in this study	
*Acosmia maotiania*	RCCBYU 10234	Figure 12.3b in [23] Figure 17.16a in [25]. Figure 3 in this study	Two individuals on one slab
*Acosmia maotiania*	RCCBYU 10235	Figure 12.3c in [23] Figure 17.16b in [25] Figure 2 in this study	
*Acosmia maotiania*	YKLP 11410	Figure 4 in this study	
New taxon 1	RCCBYU 10236	Figure 12.3d in [23] Figure 17.16c in [25] Additional File 1 in this study	Labelled *Acosmia maotiania* in [23, 25]. Distinguished here by pharyngeal and cuticular morphology, see Additional File 1
New taxon 1	YKLP 11411	Additional File 1 in this study	Labelled as *Acosmia maotiania* in YKLP collection. Distinguished here by pharyngeal and cuticular morphology, see Additional File 1

Full list of known specimens of *Acosmia maotiania.* ELRC accessioned at Nanjing Institute of Geology and Palaeontology, Chinese Academy of Sciences. RCCBYU or YKLP accessioned at Yunnan Key Laboratory for Palaeobiology, Yunnan University

#### Locality and stratigraphy

**Fig. 1:**
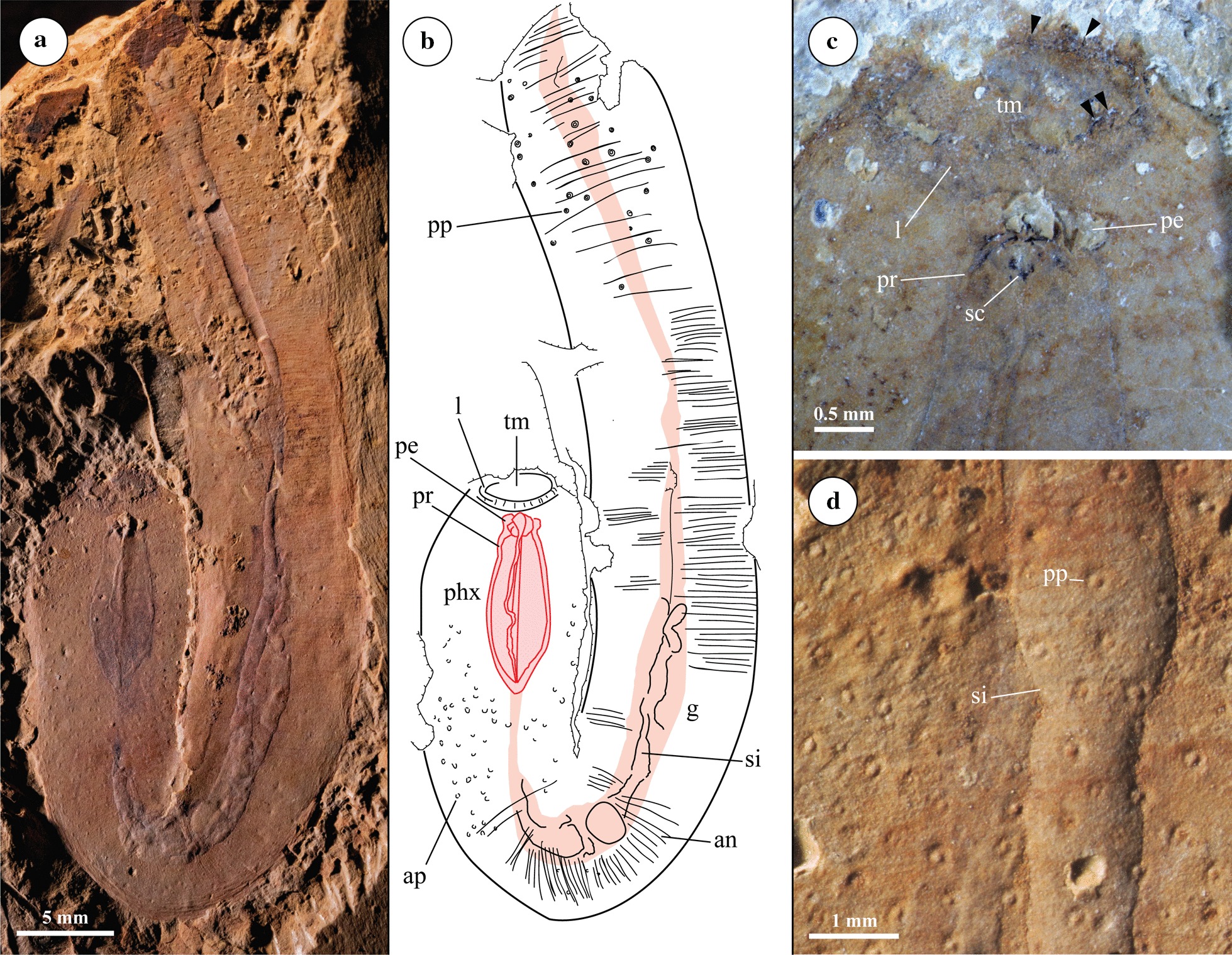
RCCBYU 10233 *Acosmia maotiania* in lateral orientation. **a** Polarized light photograph. **b** Digitised Camera Lucida. **c** Close up of the oral and pharyngeal morphology, showing the mouth, lip, and pharyngeal ridges connecting to the associated elements in the anterior portion of the pharynx. Black triangles indicate possible oral spines. **d** Close up of the posterior trunk cuticle, showing posterior papillae and infilled gut. *an* annulations, *ap* anterior papillae, *g* gut, *l* lip, *pe* pharyngeal elements, *phx* pharynx, *pp* posterior papillae, *pr* pharyngeal ridges, *sc* sclerotized tissue, *si* sediment infill, *tm* terminal mouth. Extent of the gut (pink) and pharynx (red) highlighted

**Fig. 2:**
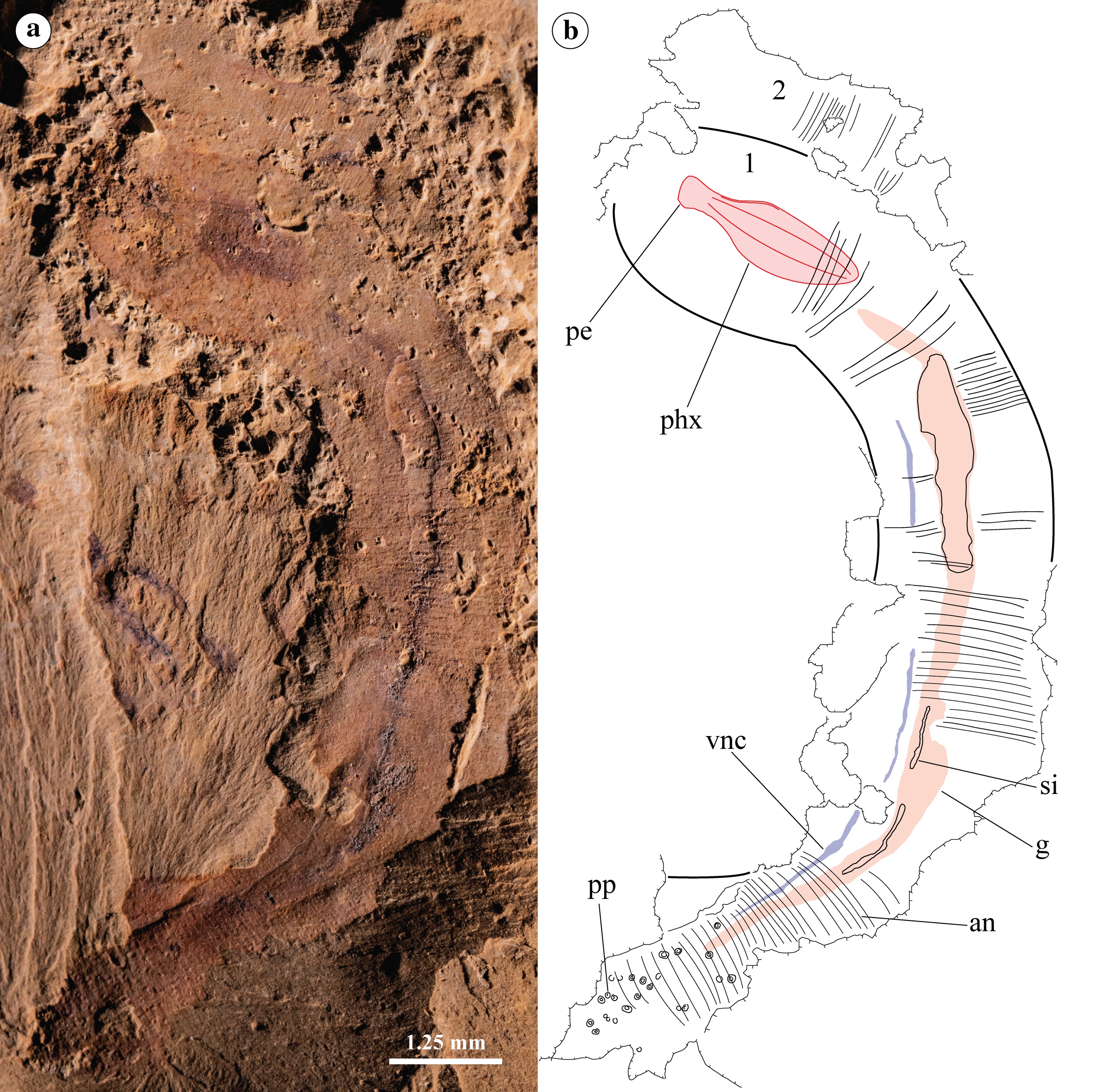
RCCBYU 10235 *Acosmia maotiania* in lateral orientation. **a** Polarized light photograph. **b** Digitised Camera Lucida. 1 = first individual, 2 = second individual (unidentified), *vnc* ventral nerve cord, other abbreviations as in Fig. 1. Extent of the gut (pink) and pharynx (red) highlighted

**Fig. 3:**
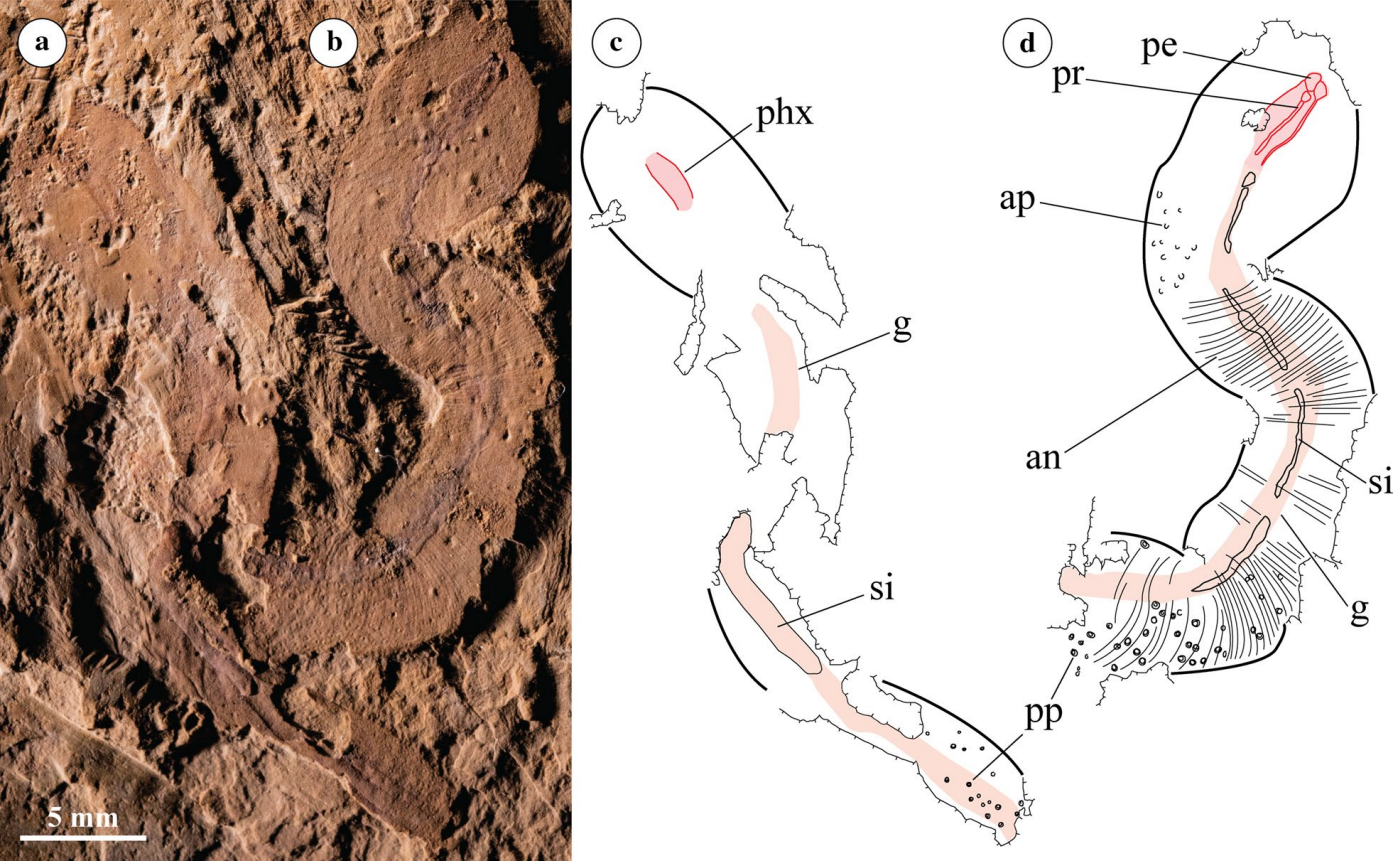
RCCBYU 10234 two individuals of *Acosmia maotiania* in lateral orientation. **a** Polarized light photograph of individual “a”. **b** Polarized light photograph of individual “b”. **c** Digitised Camera Lucida of individual “a”. **d** Digitised Camera Lucida of individual “b”. Abbreviations as for Fig. 1. Extent of the gut (pink) and pharynx (red) highlighted in both individuals

**Fig. 4:**
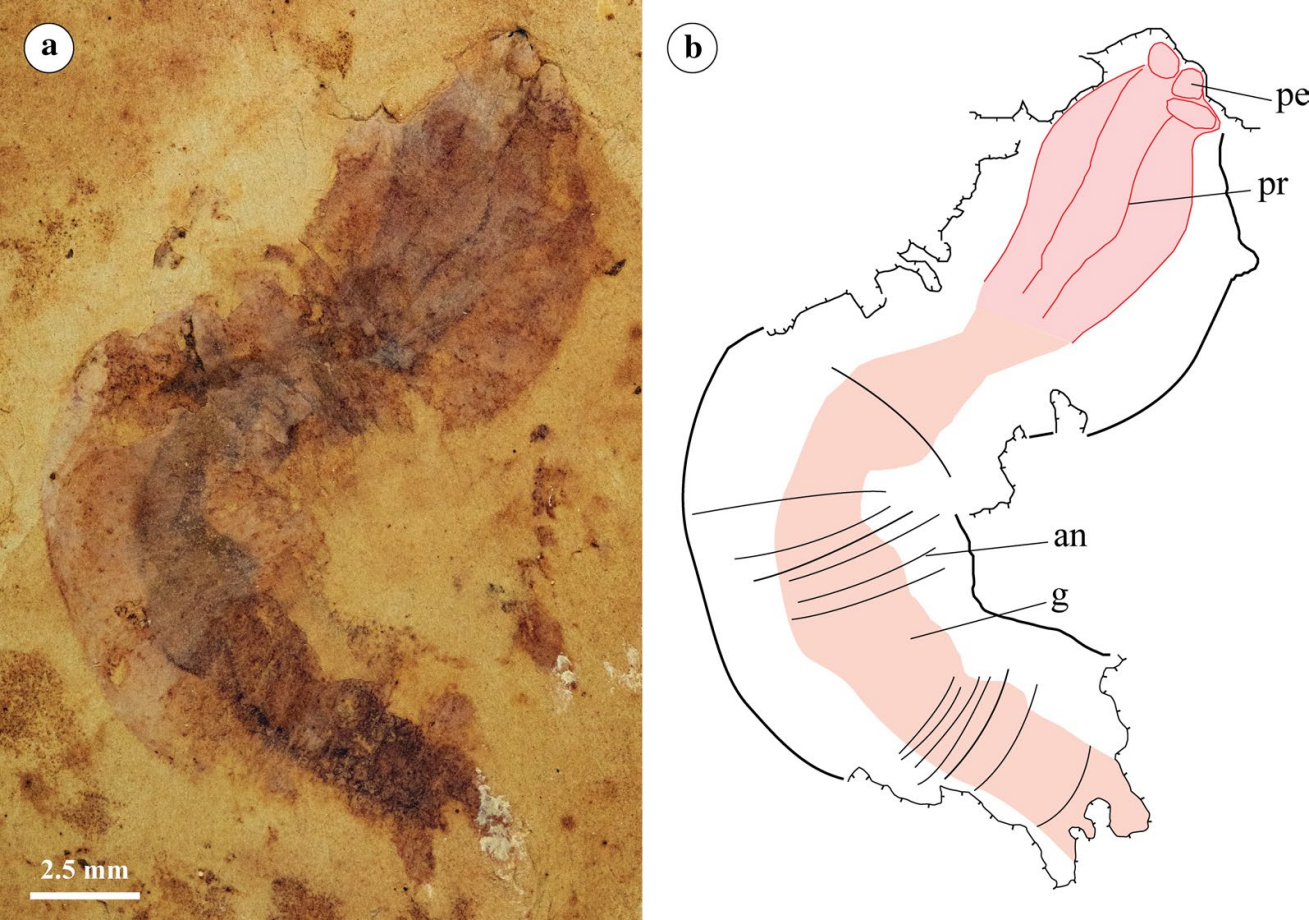
YKLP 11410 *Acosmia maotiania* in lateral orientation. **a** Polarized light photograph. **b** Digitised Camera Lucida. Abbreviations as for Fig. 1. Extent of the gut (pink) and pharynx (red) highlighted

Chengjiang Biota, Yunnan Province, People’s Republic of China. Chiungchussu Formation, Yu’anshan Member (*Eoredlichia*-*Wutingaspis* Biozone), Cambrian Series 2, Stage 3 [25]. Holotype material from Maotianshan section was not figured here [20]. Of material figured here (Figs. 1, 2, 3, 4 and Additional File 1), RCCBYU 10233–10236 from Maotianshan section in Chengjiang County, and YKLP 11410–11411 from Jianshan section in Haikou County.

#### Emended diagnosis

Body cylindrical, subdivided into anterior proboscis and posterior trunk. Proboscis slightly wider than trunk medially, separated by a slight constriction. Proboscis ornamented with conical papillae in positive relief distal to the mouth (anterior papillae). Trunk finely annulated, with button-like papillae set in pits at the posterior end (posterior papillae). Alimentary canal comprises a wide terminal mouth, a muscular barrel-shaped pharynx, and a broad through gut. Four parallel longitudinal ridges adorn the pharynx, each connecting to an anterior pharyngeal element.

### Description

This worm is relatively large, up to 100 mm long and 8 mm wide. The specimens are typically flattened and preserved in a light brown colour. Specimens studied here depicted in Figs. 1, 2, 3 and 4.

#### Mouth

The mouth is located in an anterior terminal position. Previous descriptions reported circumoral hooks [20, 23, 25]. RCCBYU 10233 preserves the mouth most clearly, showing its great circumference and a thick “lip” (labelled “l” Fig. 1), which is also clear in the holotype (ELRC 51001) figured by Chen and Zhou [20]. Dark pigment irregularly encircling the inner margin of the “lip” in RCCBYU 10233 (Fig. 1c) possibly depicts a few spiniform projections previously interpreted as hooks, but unambiguous circumoral structures are not identified.

#### Anterior proboscis

The proboscis extends about a quarter of the length of the animal, and is widest medially with a slight posterior tapering separating it from the trunk. The proboscis lacks annulation and is ornamented with conical papillae in positive relief (Fig. 1a, b, labelled “ap”). This ornamentation lacks a radial arrangement, and differs in preservation style to the dark spines and compressed spots exhibited on the scalid-covered introverts of Chengjiang scalidophorans [27]. Additionally, this ornamentation appears only in the posterior region of the proboscis and so does not surround the mouth.

#### Posterior trunk

The trunk is cylindrical and finely annulated with approximately 60 annuli per cm. The posterior papillae are button-like rather than conical, occur only in the terminal region of the trunk (Figs. 1, 2, 3, labelled “pp”), and are distinctly set in pits. The spacing and arrangement of the papillae is irregular.

#### Pharynx

The pharynx is broad and muscular, with prominent marginal ridges preserved in positive relief in RCCBYU 10233 and RCCBYU 10234b (Figs. 1a, b and 3b, d, labelled “pr”). These ridges run the length of the pharynx in a parallel longitudinal orientation and are each connected to an individual anterior element. These pharyngeal elements are poorly defined in shape but are consistent in position. They are preserved in relief in RCCBYU 10233, RCCBYU 10234b and RCCBYU 10235 (Figs. 1a–c, 3b, d, 4). Four sets of ridges and elements can be discerned in RCCBYU 10233, with one medially positioned ridge/element overlapping another, whilst two lateral ridge/elements are also clear (Fig. 1a-c, labelled “pr”/”pe”). RCCBYU 10233 exhibits patches of black carbonaceous film on the elements/ridges indicating a degree of sclerotization (Fig. 1c, labelled “sc”). The pharynx was described as retracted by Chen and Zhou [20], and is “retracted” in all specimens reported here as well. However, this assumption relies on the assumption that *Acosmia* is a priapulan—there is otherwise no evidence of pharyngeal eversibility in *Acosmia*.

#### Alimentary canal

Following on from the terminal mouth and muscular pharynx, the intestine flows the length of the body. The intestine widens in the posterior trunk compared to the anterior proboscis and shows three-dimensional sediment infilling throughout (Figs. 1, 2, 3, labelled “si”).

#### Nerve cord

An inferred ventral nerve cord is visible as a continuous dark compression, distinctly offset from the gut in RCCBYU 10235 (Fig. 2, labelled “vnc”). Neural tissues in the Chengjiang Biota are well known among arthropods [30–34], and have also been reported in priapulans [35]. The veracity of these interpretations has recently been supported by similar reports of temporally contemporaneous neural preservation in North American deposits [36].

### Phylogenetic analyses

**Fig. 5:**
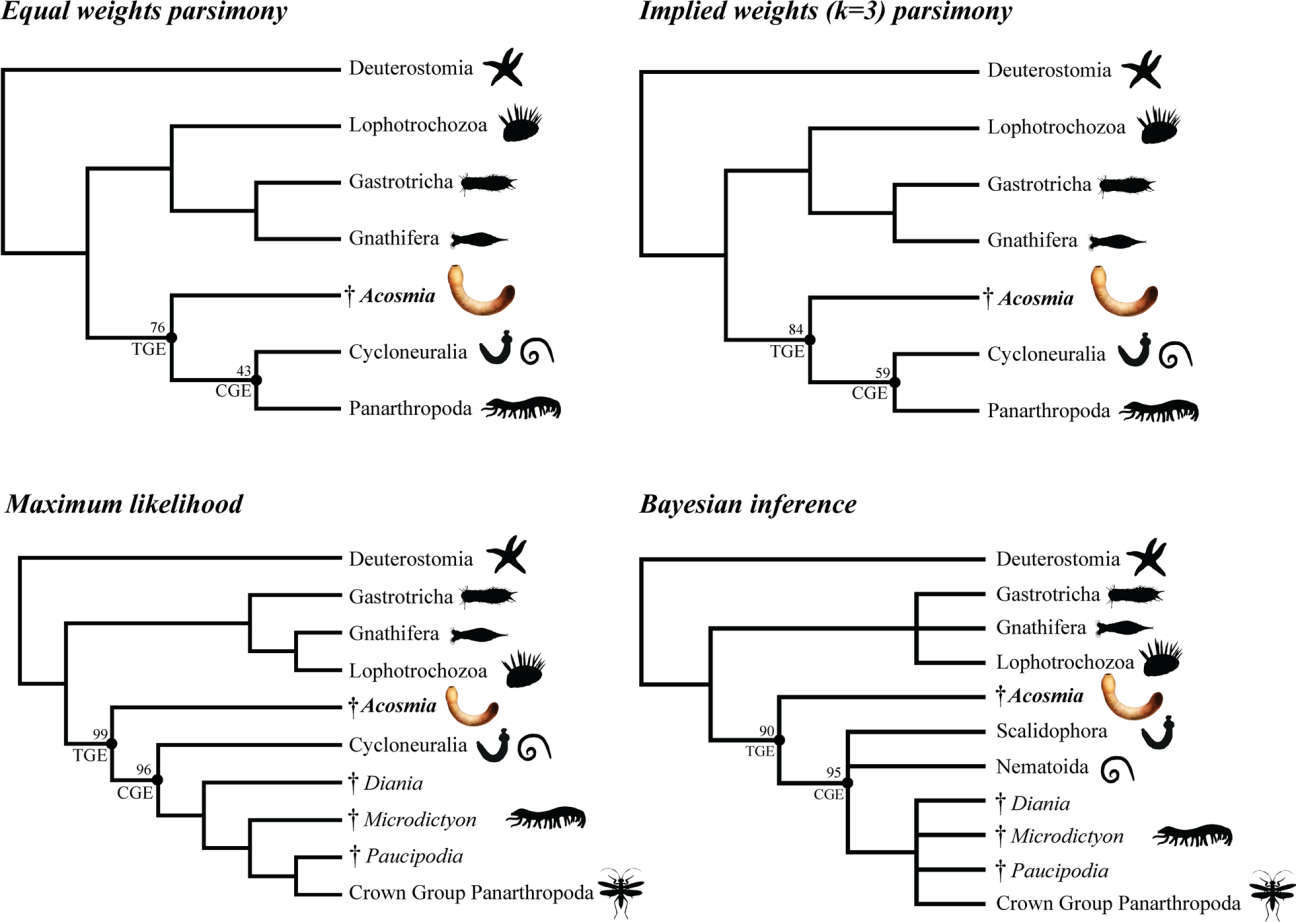
Summary of tree searches, showing simplified topology of each optimality criterion. *TGE* total group Ecdysozoa, *CGE* crown group Ecdysozoa. See Methods for explanation of nodal support values. See supplementary material for full topologies. Silhouettes from phylopic.org. *Acosmia* life reconstruction credited to Franz Anthony

All phylogenetic analyses recovered *Acosmia* as the sister group to Panarthropoda + Cycloneuralia, or sister group to a polytomy comprising Panarthropoda, Nematoida and Scalidophora (Fig. 5 and Additional files 2, 3, 4, 5). As such, *Acosmia* is resolved within the ecdysozoan stem group. Therefore, the ecdysozoan crown group can be defined as the last common ancestor of Panarthropoda + Cycloneuralia and all of its descendants. All other known ecdysozoans are therefore within the crown group. When coding the putative spines of *Acosmia* as circumoral structures (character 185, Additional File 6) rather than coding for their absence, the position of *Acosmia* as sister group to other Ecdysozoa is stable under equal and implied weights parsimony, maximum likelihood and Bayesian inference.

Spiralia was recovered as the sister group to Ecdysozoa (*Acosmia* + (Cycloneuralia + Panarthropoda)). Within Spiralia, the sister group relationships between some phyla (i.e. Entoprocta) were variable across optimality criteria, but the basic tree shape conforms to that of Vinther and Parry [37] from which the dataset is partly derived (additional data files 2, 3, 4, 5). A basal split between a clade comprising Gnathostomulida, Micrognathozoa, Rotifera and Chaetognatha (i.e. Gnathifera) and a clade similar to Lophotrochozoa comprising Nemertea, Entoprocta, Bryozoa, Brachiopoda Phoronida, Platyhelminthes, Annelida and Mollusca was almost constant. Only Gastrotricha did not conform to this split consistently. Gastrotricha was recovered as the sister group to Gnathifera in all parsimony analyses (Additional Files 2, 3), the sister group to other Spiralia using maximum likelihood (Additional File 4), and unresolved in a basal spiralian polytomy with Gnathifera and the Lophotrochozoa-like clade using Bayesian inference (Additional File 5).

Parsimony (Additional Files 2, 3) and maximum-likelihood (Additional File 4) tree searches resolved Cycloneuralia as monophyletic, whereas Bayesian inference (Additional File 5) recovered a polytomy comprising Nematoida, Scalidophora and Panarthropoda. Strict consensuses of equal and implied weights parsimony tree searches each recovered a polytomy comprising Nematoda, Nematomorpha and Scalidophora, whereas maximum likelihood and Bayesian inference recovered Nematoida as a monophylum. The relationships between scalidophorans sampled were mostly unresolved by parsimony and Bayesian inference, though all analyses recovered a sister group relationship between *Priapulus* and *Xiaoheiqingella* (i.e. Priapulida), between Nanaloricidae and Pliciloricidae (i.e. Loricifera), and between *Maotianshania* and *Cricocosmia* + *Tabelliscolex* (i.e. Palaeoscolecida). Maximum likelihood additionally recovered Priapulida as sister group to Kinorhyncha + Loricifera, with three successively branching lineages comprising the scalidophoran stem group. From stem to crown, these comprised *Corynetis* + *Louisella* (i.e. Miskoiidae, also recovered by Bayesian inference and equal weights), Palaeoscolecida, and a clade comprising *Eximipriapulus*, *Ottoia*, *Eopriapulites* and *Eokinorhynchus*.

The topology of Panarthropoda was relatively labile across optimality criteria. The lobopodians *Diania*, *Paucipodia* and *Microdictyon* were resolved in stem group Panarthropoda by maximum likelihood (Additional File 4) and Bayesian inference (Additional File 5). However, these taxa resolved within the onychophoran total group using implied weights parsimony (Additional File 3), and in a basal panarthropod polytomy along with the lobopodian *Aysheaia*, total group Arthropoda, and a clade comprising Tardigrada + total group Onychophora using equal weights parsimony (Additional File 2). Tardigrada was resolved as sister group to other panarthropods using implied weights, but was recovered as the sister group to total group Onychophora in all other optimality criteria. The stem lineage of Arthropoda was consistent across optimality criteria, comprising (in stemward to crownward order) *Megadictyon, Kerygmachela, Pambdelurion*, *Hurdia,* and *Fuxianhuia.* The exception was implied weights, which also included *Aysheaia* as the most basal member of total group Arthropoda. The stem lineage of Onychophora was less stable across optimality criteria, but always included Luolishaniidae, *Hallucigenia, Onychodictyon* and *Cardiodictyon.*

### Ancestral character state reconstructions

**Fig. 6:**
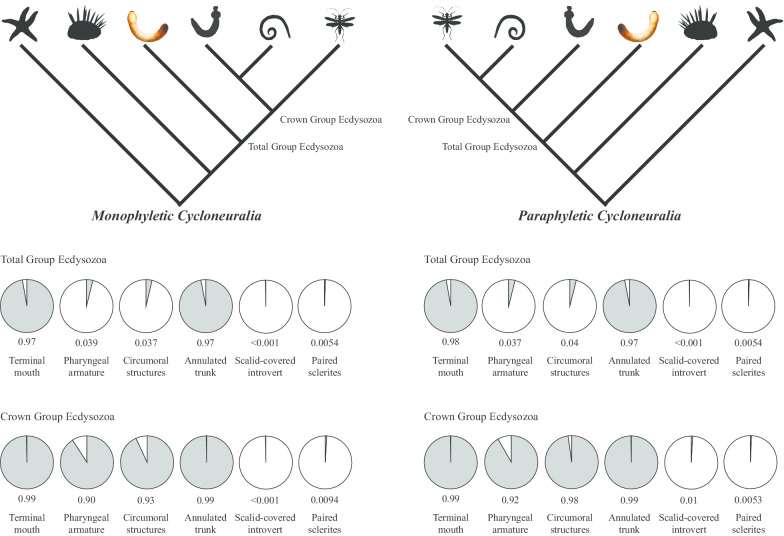
Visualization of ancestral character state reconstructions. *TGE* total group Ecdysozoa, *CGE* crown group Ecdysozoa. Percentages in pie charts represent posterior probability of the state of presence (1) for that character. Silhouettes from phylopic.org. *Acosmia* life reconstruction credited to Franz Anthony

Ancestral state reconstructions calculated here constitute the probability of the state of absence (0) vs the probability of the state of presence (1) for six key morphological characters (Tables 2 and 3, and Fig. 6) at the ecdysozoan total group node, the ecdysozoan crown group node, Cycloneuralia, Nematoida + Panarthropoda, Scalidophora, and Panarthropoda. For example, the probability that the ecdysozoan crown group ancestor had a character state of 1 (presence) for the character “adult terminal mouth” under a monophyletic Cycloneuralia topology (character 41, see Additional File 6) is 0.998708, whereas the probability that it had a character state of 0 (absence) for this character is 0.001292. Therefore, it is more probable (than not) that the crown group ancestor of Ecdysozoa had an adult terminal mouth, based on the distribution of that character state in the topology and the model of morphological evolution employed by the analysis. The latter is the MK model, analogous to basic principles of Jukes Cantor 69, i.e., equal state transitions in all directions [38].

**Table 2:** Ancestral character state reconstructions for monophyletic Cycloneuralia topology

Character	Total group Ecdysozoa (PP)	Crown group Ecdysozoa (PP)	Cycloneuralia (PP)	Scalidophora (PP)	Panarthropoda (PP)	Present in *Acosmia* ?
Terminal mouth	0 = 0.025844 1 = 0.974156	0 = 0.001292 1 = 0.998708	0 = 0.000311 1 = 0.998708	0 = 0.000112 1 = 0.999888	0 = 0.001364 1 = 0.998636	Yes
Pharyngeal armature	0 = 0.960884 1 = 0.039116	0 = 0.099041 1 = 0.900959	0 = 0.074002 1 = 0.925998	0 = 0.001654 1 = 0.998346	0 = 0.029938 1 = 0.970062	No
Circumoral structures	0 = 0.962664 1 = 0.037336	0 = 0.066616 1 = 0.933384	0 = 0.005602 1 = 0.994398	0 = 0.000135 1 = 0.999865	0 = 0.051245 1 = 0.948755	No (spines possibly present)
Annulated trunk	0 = 0.028351 1 = 0.971649	0 = 0.001323 1 = 0.998677	0 = 0.000592 1 = 0.999408	0 = 0.009102 1 = 0.990898	0 = 0.000251 1 = 0.999749	Yes
Scalid covered introvert	0 = 0.999969 1 = 0.000031	0 = 0.999636 1 = 0.000364	0 = 0.995771 1 = 0.004229	0 = 0.003437 1 = 0.996563	0 = 0.999987 1 = 0.000013	No
Paired sclerites	0 = 0.994583 1 = 0.005417	0 = 0.990559 1 = 0.009441	0 = 0.996393 1 = 0.003607	0 = 0.993516 1 = 0.006484	0 = 0.955548 1 = 0.044452	No

Values of ancestral character state reconstructions. 0 = absence of character, 1 = presence of character, PP = posterior probability

**Table 3:** Ancestral character state reconstructions for paraphyletic Cycloneuralia topology

Character	Total group Edysozoa (PP)	Crown group Ecdysozoa (PP)	Scalidophora (PP)	Panarthropoda + Nematoida (PP)	Panarthropoda (PP)	Present in *Acosmia* ?
Terminal mouth	0 = 0.024837 1 = 0.975163	0 = 0.000858 1 = 0.999142	0 = 0.000128 1 = 0.999872	0 = 0.000288 1 = 0.999712	0 = 0.001408 1 = 0.998592	Yes
Pharyngeal armature	0 = 0.962564 1 = 0.037436	0 = 0.082954 1 = 0.917046	0 = 0.002036 1 = 0.997964	0 = 0.068972 1 = 0.931028	0 = 0.022113 1 = 0.977887	No
Circumoral structures	0 = 0.959800 1 = 0.040200	0 = 0.021808 1 = 0.978192	0 = 0.000288 1 = 0.999712	0 = 0.006146 1 = 0.993854	0 = 0.010114 1 = 0.989886	No (spines possibly present)
Annulated trunk	0 = 0.027979 1 = 0.972021	0 = 0.002343 1 = 0.997657	0 = 0.011783 1 = 0.988217	0 = 0.000535 1 = 0.999465	0 = 0.000203 1 = 0.999797	Yes
Scalid covered introvert	0 = 0.999838 1 = 0.000162	0 = 0.989892 1 = 0.010108	0 = 0.003788 1 = 0.996212	0 = 0.999403 1 = 0.000597	0 = 0.999986 1 = 0.000014	No
Paired sclerites	0 = 0.994518 1 = 0.005482	0 = 0.994662 1 = 0.005338	0 = 0.993516 1 = 0.006484	0 = 0.995261 1 = 0.004739	0 = 0.957987 1 = 0.042013	No

Values of ancestral character state reconstructions. 0 = absence of character, 1 = presence of character, PP = posterior probability

In order to account for topological uncertainty within Ecdysozoa (see “Methods”—“Topology sensitivity tests”), ancestral state reconstruction analyses were performed on two alternate trees. (1) Monophyletic Cycloneuralia: Panarthropoda (Nematoida + Scalidophora), as recovered by morphology (as in most of the analyses herein); (2) Paraphyletic Cycloneuralia: Scalidophora (Nematoida + Panarthropoda), as in most phylogenomic analyses [39–41], although mostly lacking data for one or more phyla. Posterior probabilities of ancestral character states were affected by the two contrasting topologies by small amounts in all cases. For some characters, the difference between the two topological hypotheses were negligible: the presence of a terminal mouth and an annulated trunk yielded a posterior probability of > 0.99 pp for both mono- and paraphyletic Cycloneuralia at the crown group node, and > 0.97 pp for the total group nodes, and similarly high for Cycloneuralia, Scalidophora, Nematoida + Panarthropoda and Panarthropoda. Similarly, the presence paired sclerites remained at < 0.01 pp for the total and crown group nodes under both topologies, and was at < 0.05 pp for Scalidophora, Panarthropoda and Nematoida + Panarthropoda. The probability of presence of pharyngeal armature and circumoral structures remained > 0.90 pp across both analyses for the crown group node, but with small increases using the paraphyletic Cycloneuralia topology compared to monophyletic. These two characters however yielded high probability for absence at the total group node (0 ≥ 0.95 pp), but remained high probability for presence in Cycloneuralia, Scalidophora, Nematoida + Panarthropoda and Panarthropoda. The probability of presence of a scalid covered introvert was extremely low across both analyses at the total and crown group nodes (< 0.01 pp), but high for Scalidophora (> 0.99 pp).

## Discussion

### Taphonomic research supports the basal position of *Acosmia*

The coding of ecdysozoan fossils into the phylogenetic matrix was informed by taphonomic decay studies of extant taxa [42–44]. This was necessary to deduce the designation of character states as unknown (?) or absent (0), and to account for the possibility of “stem-ward slippage”—the phenomena whereby fossils appear erroneously primitive due to biases towards plesiomorphic character preservation in their decay process. Most significantly for our interpretations, the decay process in *Priapulus* was taken into account [29] when designating the character states of *Acosmia*—which was previously regarded as a priapulan [20]. Decay experiments showed that scalids and pharyngeal armature were among the most recalcitrant of all anatomical structures in the decay of *Priapulus*. These morphological features do not occur in *Acosmia,* but other cuticular structures designated highly recalcitrant by Sansom [29] do occur in *Acosmia* such as annulations and trunk papillae (though probably not directly homologous to the anterior and posterior papillae of *Acosmia*). This shows that the cuticular anatomy of *Acosmia* has been preserved in sufficient fidelity for scalids and pharyngeal teeth to be present if they occurred. As they do not occur in any known specimen, their absence in *Acosmia* is likely to be genuine and not the result of a taphonomic bias. Furthermore, Sansom [29] found no evidence for stem-ward slippage among priapulans when decay-informed character coding was employed, as only the most recalcitrant characters (i.e. those pertaining to the cuticle) appear to be phylogenetically informative. Murdock et al. [45] found this was also the case in the other side of the cycloneuralian-panarthropod dichotomy, employing similar methods on onychophorans to the same result. Therefore, stem-ward slippage (i.e. decay bias against apomorphies like scalids, pharyngeal armature etc.) is not considered to be as problematic in ecdysozoan phylogeny as it is in early vertebrate phylogeny for example [46, 47]. As such, experimental decay research supports *Acosmia’*s basal phylogenetic position.

### Lifestyle of the ecdysozoan worm *Acosmia maotiania*

Taphonomically informed phylogenetic analyses according to four alternative optimality criteria resolved *Acosmia* as a stem lineage ecdysozoan (Fig. 5). *Acosmia* therefore represents among the only direct palaeontological models to hypothesise how ecdysozoans might have originated and diversified. As such, it is necessary to consider the ecology of *Acosmia*. *Acosmia* is a little known Chengjiang fossil, appearing only in successive review-style compilations of the fauna [20, 22–25], and is listed as a priapulan each time—though authors are consistently doubtful of the priapulan affinity. The inference of burrowing behaviour is based on the aspect of preservation in some specimens in a “U” shape (e.g. Figures 1, 2), the idea being that *Acosmia*, with its infilled through gut and muscular pharynx, had a deposit-feeder lifestyle in the upper reaches of the muddy sediment like a lugworm in a U-shaped burrow. Assuming this reconstruction is accurate, it could be inferred that the acquisition of pharyngeal armament (i.e. teeth [14]) facilitated the transition from deposit feeding by suction in *Acosmia*–like ecdysozoans to predation in cycloneuralians and lobopodians using teeth and stylets to capture and process prey items in the sediment. However, this would also rely on the assumption that *Acosmia* represents a typical member of the ecdysozoan stem-lineage and had not adapted to a deposit feeding lifestyle independently.

### Ancestral ecdysozoan characters are constrained by *Acosmia*

**Fig. 7:**
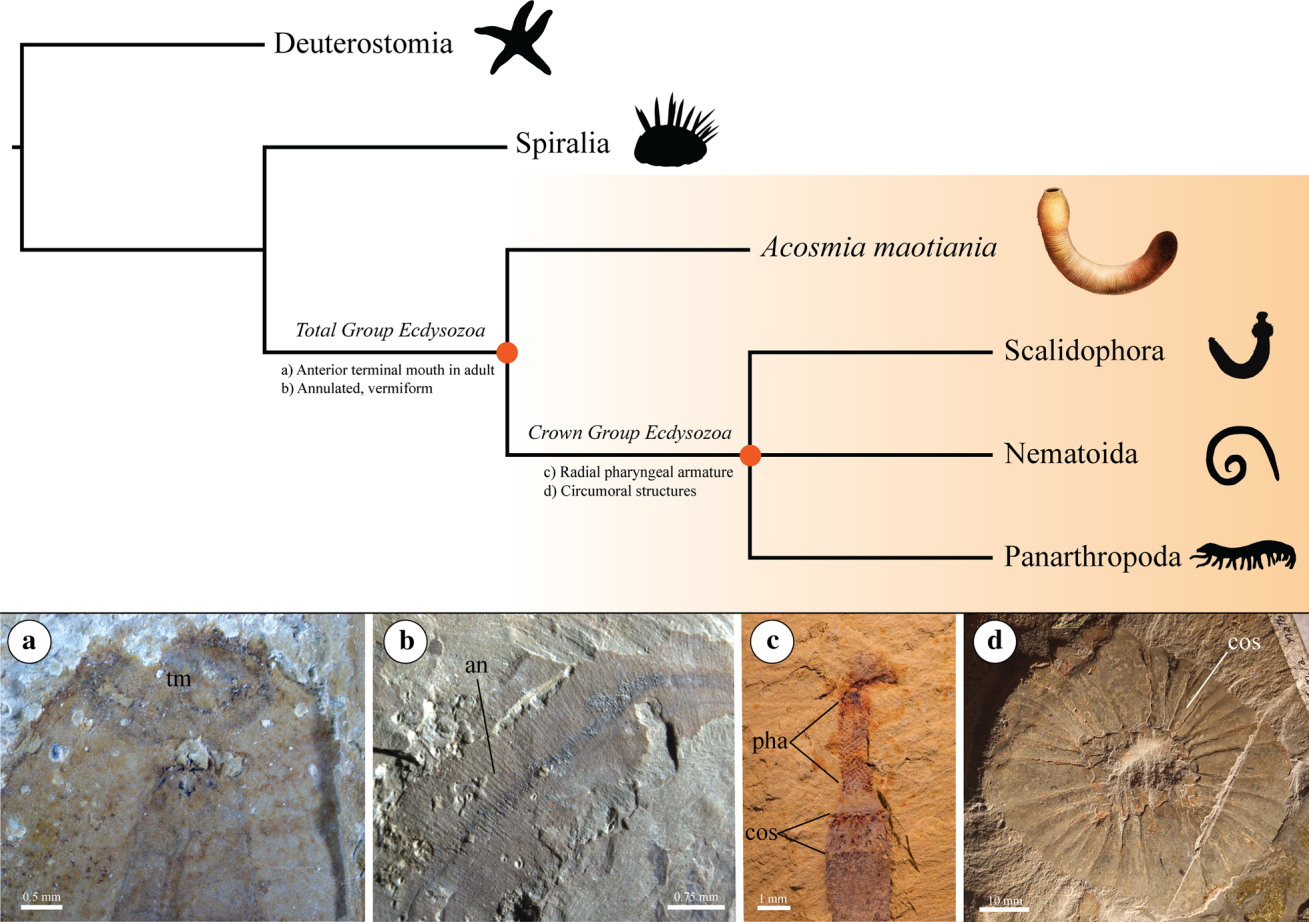
Optimisation of well-supported ancestral characters on topology, with fossil exemplars. **a** Anterior terminal mouth of *Acosmia maotiania* (RCCBYU 10233) in lateral orientation (normal light). **b** Annulated trunk of *Acosmia maotiania* (RCCBYU 10235) in lateral orientation (polarized, low angle). **c** Circumoral structures (scalids) and pharyngeal armature (teeth) of *Cricocosmia jinningensis* (YKLP 11412) in lateral orientation (polarized, low angle). **d** Circumoral structures (plates) of *Peytoia nathorsti* (USNM 57538) in ventral orientation (polarized, low angle). *cos* circumoral structures; *pha* pharyngeal armature. Photograph D credited to Allison Daley, all others to Richard Howard. Silhouettes from phylopic.org. *Acosmia* life reconstruction credited to Franz Anthony

Characters selected for ancestral state reconstruction constituted traits that might be inferred as ecdysozoan plesiomorphies from studies of crown group taxa—though of course this is dependent on the topology under consideration. Characters considered plesiomorphies are optimised in Fig. 7.

1. Adult terminal mouth: In contrast to other bilaterian groups, an adult terminal mouth has been proposed as ancestral for Ecdysozoa [19, 48, 49]. Extant arthropods and onychophorans lack this character (in addition to some nematodes and some heterotardigrades)—but the fossil record indicates that this is the result of secondary modification [19]. Most non-arthropod Cambrian ecdysozoans (e.g. many lobopodians, archaeopriapulids, palaeoscolecids) possess an anterior terminal mouth in their presumed adult form like several extant groups (i.e. all extant scalidophorans, most nematoids, most tardigrades), and taxa lacking this character occupy derived phylogenetic positions within their respective lineages. For example, the stem group arthropods *Pambdelurion* and *Hurdia* have ventral mouths. However, these taxa are located crownward of arthropod taxa with terminal mouths such as *Megadictyon*, and so the ventral orientation is inferred to be secondary. As this character is present in *Acosmia* and highly probable to have been present at both the total group and crown group ecdysozoan nodes (pp ≥ 0.97 for both nodes and both topological hypotheses, see Tables 2 and 3), an anterior terminal mouth is highly probable to be ancestral for Ecdysozoa.
2. Pharyngeal armature: Ecdysozoans are not the only protostomes with prominent pharyngeal structures. Various spiralian groups exhibit jaw and tooth like structures within their pharynxes, notably the Gnathifera [50]. Gnathifera is supported by phylogenomics as a clade within Spiralia containing Rotifera + Acanthocephala (Syndermata), Gnathostomulida, Micrognathozoa, and possibly Chaetognatha [40, 41, 51, 52]—the inclusion of which receives additional support from Cambrian fossils [37]. However, the pharyngeal structures of gnathiferans are clearly distinct from those of ecdysozoans. Gnathiferan pharynxes are equipped with bilaterally symmetrical and complex jaw apparatuses [50], which do not resemble the radially arranged teeth and stylets of extant and fossil ecdysozoans. As such, they were not scored as equivalent structures here in the phylogenetic character matrix. Ecdysozoan pharyngeal armature varies by group and was scored on a simple absence or presence basis in the character matrix under the assumption that these structures are homologous based on their consistent position ornamenting the cuticle of the pharynx, and their typically radial symmetry.
  With some exceptions (extant Onychophora for example), the pharynxes of ecdysozoans are commonly armed with teeth, spines or stylets etc. Little has been done to characterise the homology of these structures across the diversity of Ecdysozoa. However, the discovery of pharyngeal teeth of a similar nature between Cambrian cycloneuralians (e.g. [53].) and Cambrian panarthropods [14, 18, 54–56] has promoted the idea that these represent an ancestral character for Ecdysozoa—especially given the presence of radial tooth-like structures in some living panarthropods [57, 58]. Priapulans often exhibit cuspidate pharyngeal teeth (e.g. *Halicryptus spinulosus* [59]) which are arranged in rings of five-fold symmetry (quincunxes). These are mirrored in some exceptionally preserved priapulan-like fossils such as *Ottoia prolifica* [53] from the Burgess Shale. Other less obviously priapulan-like fossil scalidophorans exhibit pharyngeal teeth that are more simple and spinose, but are similarly radial in their arrangement—for example the phosphatic microfossil *Eokinorhynchus rarus* [13, 60] from the Fortunian of Sichuan Province, China. Kinorhynchs and loriciferans lack pharyngeal teeth but are themselves armed with specialised radial pharyngeal armature. Nebelsick [61] reported three quincunxes of articulating pharyngeal stylets in the cyclorhagid *Echinoderes capitatus*, and determined they were sensory in function. Loriciferans also bear stylets, but they are oral features associated with the extensible buccal tube rather than the pharynx [62]. Whether this represents a migration of an ancestrally pharyngeal structure is unknown. However, nanaloricid loriciferans at least bear a triradial pattern of rows of thickened cuticular elements known as placoids [62]. The topologies presented here would suggest that the pharyngeal armament of kinorhynchs and loriciferans represent derived morphologies, especially given the similarity of priapulan teeth to those of some panarthropods [18, 55].
  Nematoid pharynxes are more problematic to interpret in an evolutionary sense, as the fossil record of the group is limited to comparatively younger crown group taxa. The oldest nematoid fossil is *Palaeonema phyticum* [63], which is comparable to some extant groups of nematodes. Nothing is known about the nematoid stem group. Nematodes commonly bear stylets associated with the pharynx—especially plant parasites, but it is not clear that these structures are homologous to the teeth, stylets and placoids of other groups as they lack the radially oriented arrangement. Larval nematomorphs do show a radial pattern to their armature, but is not clear that these hexaradial piercing stylets are associated with the pharynx, the musculature of which is highly reduced in Nematomorpha [21, 26]. As such, both groups were coded uncertain (“?”) for pharyngeal armature.
  Ancestral character state reconstructions here yielded high probabilities for the presence of pharyngeal armature at the ecdysozoan crown node (> 0.90 pp for both topological hypotheses), but extremely low probabilities at the total group node (< 0.04 pp), and this character does not appear to be present in *Acosmia*. *Acosmia* does possess prominent pharyngeal structures (the ridges/elements described here), but these do not resemble the radial rings of armature exhibited by the crown group lineages. Therefore, we infer that pharyngeal armature of the kind exhibited by cycloneuralians and lobopodians is a derived character for the ecdysozoan crown group and not ancestral for Ecdysozoa.
3. Circumoral structures: Virtually all ecdysozoans, other than crown group onychophorans and arthropods crownward of radiodonts, show some form of circumoral structures. This refers to cuticular elements arranged radially around the axis of their mouth opening, resulting in an anterior plane of radial symmetry in addition to the anterior–posterior axis of bilateral symmetry. In this fashion, scalidophorans exhibit rings of scalids upon their introvert [21], nematoids may exhibit radial hooks or cephalic sensillae and setae [21, 64, 65], tardigrades exhibit a buccal ring of lamellae [58, 66], and the fossil stem groups of both arthropods and onychophorans similarly show rings of plate-like lamellae [14, 55, 56, 67]. This has been discussed previously as an ancestral character for Ecdysozoa [14], though the homology of these highly variable structures (i.e. scalids compared to radiodont oral plates) has yet to be demonstrated further.
  A recent study [68] described the introvert and pharyngeal armature of the Chengjiang worm *Mafangscolex sinensis*—Palaeoscolecida sensu stricto [17]—and postulated that a hexaradially-ornamented proboscis may be an ancestral ecdysozoan character. Similarly, the authors of a study describing *Eopriapulites sphinx*—a Fortunian stem group scalidophoran preserved as a phosphatic microfossil—made a similar hypothesis regarding the ecdysozoan groundplan [69]. This is because hexaradial symmetry is widespread among the circumoral structures of both fossil and extant Ecdysozoa (except for some Scalidophora, such as extant Kinorhyncha and Priapulida), and because the authors infer that palaeoscolecids are not stem group priapulans as reported by some analyses [17]. Yang et al. [68] estimated instead that palaeoscolecids form a paraphyletic group at the base of Ecdysozoa, and as such may reflect the ancestral condition of Ecdysozoa. Our study mostly does not controvert the findings of Yang et al. [68] or Liu et al. [69], as our phylogenetic analyses did not recover a relationship between palaeoscolecids or *Eopriapulites* and priapulans—instead recovering Palaeoscolecida and *Eopriapulites* essentially unresolved in a basal scalidophoran polytomy. As the monophyly of Scalidophora has yet to be demonstrated convincingly in phylogenomic studies, we hypothesise that palaeoscolecids such as *Mafangscolex* may possibly represent stem group Ecdysozoa as Yang et al. [68] predict, but that these worms are more crownward than *Acosmia*. When each instance of circumoral structures is coded as equivalent here on a presence or absence basis, with *Acosmia* designated absent (although noting the possible presence of hooks—see mouth in Description), the results support this character being present at the ecdysozoan crown node (> 0.93 pp), but absent at the ecdysozoan total group node (< 0.041 pp). Therefore, circumoral structures (and their inferred plesiomorphic hexaradial symmetry) are a derived character within Ecdysozoa, and not ancestral for Ecdysozoa. As such, palaeoscolecids are likely to be closer to the ecdysozoan crown group than *Acosmia*, if not within it as scalidophorans.
4. Annulation: Fossil and extant ecdysozoans typically bear an annulated trunk, that is, transverse cuticular rings along their anterior–posterior body axis. Exceptions include crown group and upper stem group arthropods [70], as well as kinorhynchs and loriciferans—which are all inferred as secondary losses due to the specialised trunk morphology of these groups. Arthropods and kinorhynchs are segmented and covered by metamerically repeated dorsal and ventral plates, whereas loriciferans are encased within a corset-like lorica. Annulations are present in *Acosmia* and are highly probable to have been present at the crown and total group nodes (> 0.97 pp for both nodes and topologies). Therefore, an annulated trunk is well supported here as an ancestral character for ecdysozoans.
5. Scalid-covered introvert: The radial spines/hooks of nematoids are of demonstrably different construction to those of scalidophorans, being comprised entirely of cuticle [26], whereas scalidophoran scalids are hollow sense/locomotive organs [21]. This form of circumoral armature was therefore recoded as absent in nematoids, as opposed to present as in Vinther and Parry [37]. As such, scalids are likely autapomorphic for Scalidophora, and they adorn a retractable anterior proboscis known as the introvert. However, this inference is impeded by the lack of phylogenomic support for the monophyly of Scalidophora. What little molecular phylogenetics has been done has resolved the Loricifera in some unconventional positions in studies using only targeted Sanger sequencing, [71, 72] but also as the sister group to Priapulida in a phylogenomic-scale study that did not include Kinorhyncha [52]. A sister group relationship between Priapulida and Kinorhyncha has been recovered by multiple studies utilizing different datasets that lacked Loricifera [39, 73, 74]. The only phylogenomic study with a taxon sample covering Priapulida, Kinorhyncha and Loricifera recovered scalidophoran paraphyly at the base of Ecdysozoa—with Loricifera as sister to Nematoda or Nematoida [40]. Scalidophoran paraphyly at the base of Ecdysozoa suggests the scalid-covered introvert could be an ancestral ecdysozoan character lost by Nematoida and Panarthropoda—an idea endorsed in some palaeontological studies [68]. Topologies employed here however all assumed monophyly of Scalidophora based on our own analyses (see Fig. 5), and all yielded an extremely low probability for presence of a scalid-covered introvert (~ 0.01 pp or less) for all nodes investigated except Scalidophora—which yielded > 0.99 pp for both topologies. As such, a scalid-covered introvert is inferred to be an autapomorphy of Scalidophora, though as discussed above, only morphological phylogenies have so far supported the monophyly of Scalidophora. Regardless, if scalidophorans do form a basal paraphyletic grade, the scalid-covered introvert would still likely represent a derived character as it does not feature in *Acosmia*—which lacks any regularly arranged proboscis armature, and the proboscis does not appear to be retractable.
6. Paired sclerites: Numerous lobopodians show metameric series of epidermal specializations above the leg pairs. These range greatly in morphology, from the hexagonally meshed ovoid plates of *Microdictyon* [75–80] to the elongated spines of *Hallucigenia* [14, 81–83], and are considered to be homologous across taxa. In addition, studies have shown the structure and composition of the modularly repeated lateral sclerites of some palaeoscolecids (such as *Cricocosmia* and *Tabelliscolex*) are highly similar to those of lobopodians [82, 84]. As such, this character has been coded as present for both groups here and in other published phylogenetic analyses [14, 85]. This suggests paired sclerites may be an ancestral ecdysozoan character, given that palaeoscolecids are distant from lobopodians in our phylogenetic analyses. However, the probabilities of paired sclerite presence at the ecdysozoan total and crown group nodes are extremely low (< 0.01 pp for both topologies). This suggests this character is of independent origin between palaeoscolecids and lobopodians—an example of the convergent evolution of metameric sclerotization in the ecdysozoan cuticle.
  However, the systematics of palaeoscolecid worms are not well resolved, and this is problematic for a hypothesis of convergence. Our study recovered a clade comprising *Maotianshania*, *Cricocosmia* and *Tabelliscolex*—elongated Chengjiang worms with armoured introverts known from soft-tissue bearing macrofossils—within a mostly unresolved Scalidophora. Harvey et al. [17] did not consider *Maotianshania, Cricocosmia and Tabelliscolex* to be “Palaeoscolecida sensu stricto”, and retrieved polyphyly of these taxa within the priapulan stem group in their most inclusive analysis. Furthermore, other studies have alluded to the polyphyly/paraphyly of palaeoscolecids by supporting homology of the paired sclerites of *Cricocosmia* and *Tabelliscolex* with those of lobopodians [82], hypothetically including *Cricocosmia* and *Tabelliscolex* within the panarthropod total group. This suggests that paired sclerites are a panarthropod apomorphy, in contrast to the results of our study. Regardless, our hypothesis that paired sclerites are not an ancestral character for Ecdysozoa remains.

## Conclusions

The early Cambrian Chengjiang taxon *Acosmia maotiania* was not a priapulan, but a worm belonging to the stem-lineage of Ecdysozoa, and represents the first fossil taxon placed as such. This provides a unique phylogenetic constraint on other Cambrian ecdysozoan fossils, and allows inferences of ecdysozoan ancestral morphology to be tested. Analyses here have shown that the ancestor of crown group Ecdysozoa shared an adult terminal mouth and annulated cuticle with *Acosmia*, but also possessed radial pharyngeal armature and circumoral structures—which *Acosmia* appears to lack. This suggests that the acquisition of radial pharyngeal armature is a derived trait of the crown group, and may have been significant in the diversification of cycloneuralians and panarthropods. However, it is important that more stem group ecdysozoans are identified in the fossil record in order to robustly test this hypothesis, with particular focus on the palaeoscolecids—which appear to be a polyphyletic group that may include stem group ecdysozoans [68]. *Acosmia* continues a theme in the study of ecdysozoan evolution over recent years [14, 55], wherein authors have recognised a precedent to the oral and pharyngeal morphology of Cambrian ecdysozoans in resolving their phylogenetic relationships and ecological roles.

## Methods

### Fossil material

Seven individuals assigned to *Acosmia maotiania* were available for study in the collections of the Yunnan Key Laboratory for Palaeobiology (out of nine known individuals, see Table 1). Specimens were examined under a Zeiss SteREO Discovery light microscope, using normal and polarized light. Specimens were photographed using a Canon EOS 750d camera equipped with a 105 mm Sigma macro lens, and a scope mounted AxioCam 5. Photographs and Camera Lucida were enhanced and digitised using Adobe Illustrator and Photoshop software (Figs. 1, 2, 3, 4, Additional File 1). Two of the seven individuals (RCCBYU 10236 and YKLP 11411) show marked differences from the other material and are determined to have been misidentified (see Additional File 1). These two specimens remain in open nomenclature here (New Taxon 1).

### Character matrix

The character matrix (included in NEXUS format; see Additional File 7) used in all analyses here comprises 62 taxa (*Acosmia*, 60 other protostomes, and a single deuterostome) scored for 185 characters. This matrix is derived from a previous study on Cambrian spiralian phylogeny [37]. We expanded this matrix to include *Acosmia maotiania* and 26 Cambrian ecdysozoan fossil taxa. 45 characters were newly scored, these derived mostly from previous studies on the phylogeny of cycloneuralians [17, 86, 87] and lobopodians [14, 83, 85, 88]. The matrix in NEXUS format and the list of scored characters are presented as Additional files 6, 7.

### Phylogenetic analyses

Phylogenetic analyses were performed to resolve the position of *Acosmia maotiania* (summarised in Fig. 5). There is considerable debate over the most appropriate model of optimality to infer morphological phylogenies [89–95]. Therefore, tree searches used four alternative optimality criteria: equal weights parsimony, implied weights parsimony, maximum likelihood and Bayesian inference.

Parsimony tree searches were conducted in TNT 1.5 [96, 97], using the New Technology tree search function. A strict consensus of four most parsimonious trees (mpt) is presented for equal character weighting, with clade support assessed by jackknife resampling [98] (Additional File 2). For implied weights (where *k* = 3), a strict consensus of four mpt is presented (Additional File 3), with clade support assessed by symmetric resampling [99]. 1000 replicates were performed for each resampling strategy under default parameters.

Maximum likelihood and Bayesian inference tree searches used the MK probabilistic model [38]. The maximum likelihood implementation was conducted in IQ-TREE [100], recovering a fully resolved topology (Additional File 4), with nodal support assessed by 300,000 ultrafast bootstrap replicates [101, 102]. The Bayesian implementation was conducted in MrBayes 3.2 [103] using the MK + gamma model. The Bayesian analysis was run until convergence of the MCMC chains after 2,000,000 generations, with convergence assessed by the average deviation of split frequencies (< 0.01), ESS scores (> 200), and PSRF values (= approx. 1.00). 25% of samples were discarded as burn in, and a majority rule consensus was output (Additional File 5).

### Ancestral state reconstructions

Ancestral state reconstructions for six morphological characters were performed on the ecdysozoan total group node, the ecdysozoan crown group node, Cycloneuralia, Nematoida + Panarthropoda, Scalidophora, and Panarthropoda (Fig. 6, Tables 2, 3). Characters selected for ancestral state reconstruction represent traits inferred as ecdysozoan plesiomorphies (ancestral characters) from studies of crown group taxa (see “Discussion”). These characters included the presence or absence of: (1) adult terminal mouth; (2) pharyngeal armature; (3) circumoral structures; (4) scalid-covered introvert; (5) annulated trunk; (6) paired sclerites.

This was carried out individually for the selected character in MrBayes by adding the “report ancstates” command to tree searches. This was employed to calculate the posterior probability of the presence (1) and absence (0) of the selected characters at the selected nodes. Analyses used the MK + gamma model, and always converged after 2–3 million generations. Average deviation of split frequencies (< 0.01), ESS scores (> 200), and PSRF values (= approx. 1.00) assessed convergence of the MCMC chains.

### Topology sensitivity tests

Morphological and molecular trees are usually incongruent regarding the clustering of Nematoida to either Scalidophora or Panarthropoda, respectively [2]. In order to account for this topological uncertainty on ancestral state probabilities, we performed our ancestral state reconstructions on two alternative topologies (see Tables 2, 3 and Fig. 6): (1) Monophyletic Cycloneuralia = Panarthropoda (Nematoida + Scalidophora); (2) Paraphyletic Cycloneuralia = Scalidophora (Nematoida + Panarthropoda). To do this, either the monophyly or paraphyly of Cycloneuralia was forced by a topology prior using the “topologypr” command in MrBayes when performing ancestral state reconstructions.

## Supplementary information

**Additional file 1: Fig. 1.** New Taxon 1 (previously referred to *Acosmia maotiania*). A) Polarized light photograph of RCCBYU 10236. B) Polarized light photograph of YKLP 11411. C) Polarized light photograph of the presumed pharynx and mouth of YKLP 11411. Abbreviations: g = gut, phx? = pharynx (presumed), ps? = pharyngeal spines (presumed), tm? = terminal mouth (presumed).

**Additional file 2: Fig. 2.** Full results of equal weights parsimony-based tree searches. Daggers indicate fossil taxa. See section Methods – phylogenetic analyses for method details.

**Additional file 3: Fig. 3.** Full results of implied weights (k=3) parsimony-based tree searches. Daggers indicate fossil taxa. See section Methods – phylogenetic analyses for method details.

**Additional file 4: Fig. 4.** Full topology of maximum likelihood tree search. Tree fully resolved and with branches transformed. Daggers indicate fossil taxa. See section Methods – phylogenetic analyses for method details.

**Additional file 5: Fig. 5.** Full topology of Bayesian inference tree search. Daggers indicate fossil taxa. See section Methods – phylogenetic analyses for method details.

**Additional file 6.** List of morphological characters used in phylogenetic analyses.

**Additional file 7.** Character matrix used in phylogenetic analyses in NEXUS format.

## Data Availability

The datasets used and/or analysed during the current study are available from the corresponding author on reasonable request. The phylogenetic data matrix is included as a downloadable NEXUS file (Additional File 7).

## Abbreviations

an: Annulations
ap: Anterior papillae
g: Gut
l: Lip
pe: Pharyngeal elements
phx: Pharynx
pp: Posterior papillae
pr: Pharyngeal ridges
sc: Sclerotized tissue
si: Sediment infill
tm: Terminal mouth
vnc: Ventral nerve cord
