# Supplementary figures and images for: Ancestral morphology of Ecdysozoa constrained by an early Cambrian stem group ecdysozoan
Source: BMC Evol Biol. 2020 Nov 23;20:156. doi: 10.1186/s12862-020-01720-6 (PMC7684930; doi:10.1186/s12862-020-01720-6)

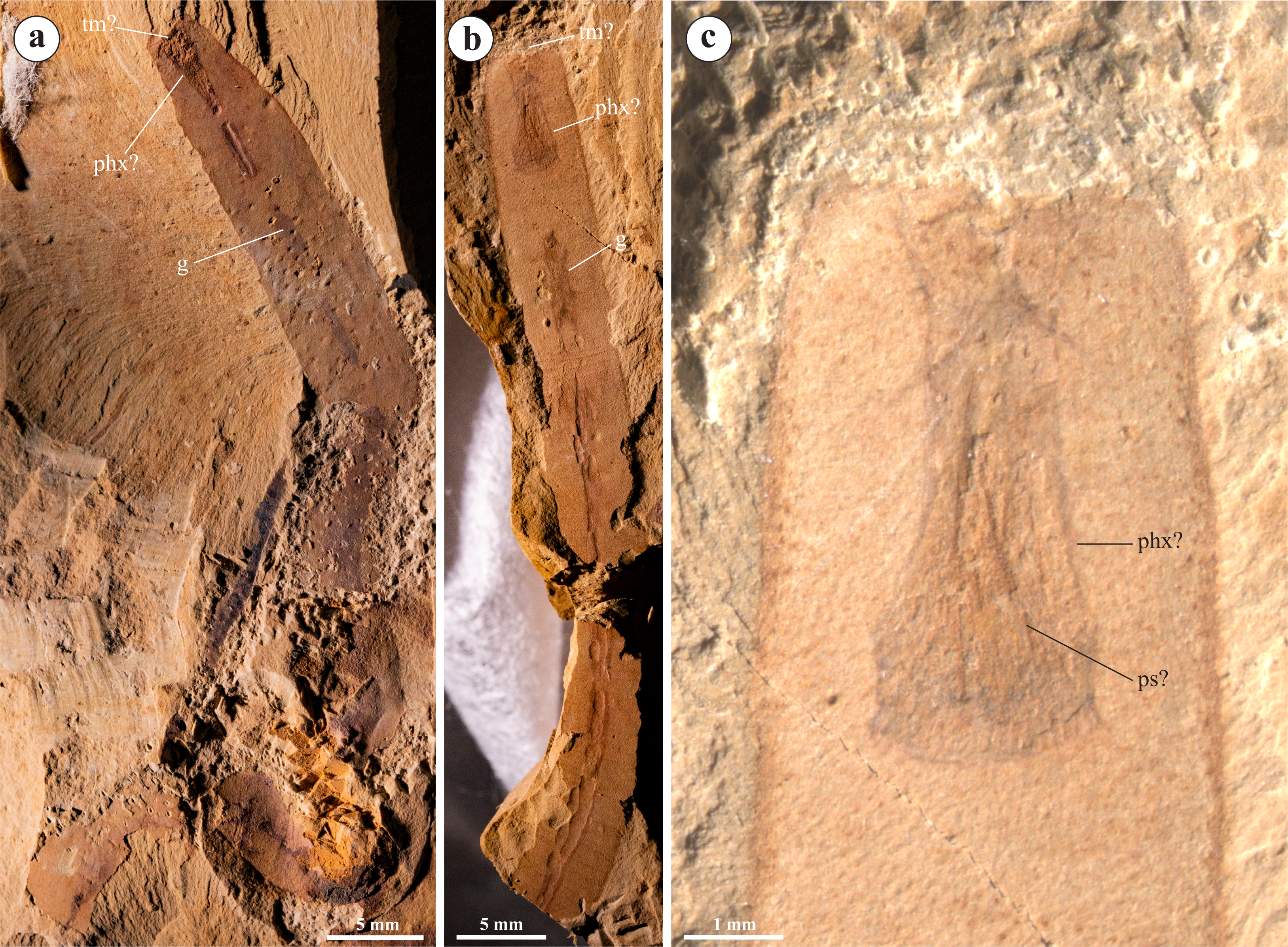

Supplement: Supplementary file 1 — Additional file 1: Fig. 1. New Taxon 1 (previously referred to Acosmia maotiania). A) Polarized light photograph of RCCBYU 10236. B) Polarized light photograph of YKLP 11411. C) Polarized light photograph of the presumed pharynx and mouth of YKLP 11411. Abbreviations: g = gut, phx? = pharynx (presumed), ps? = pharyngeal spines (presumed), tm? = terminal mouth (presumed). [file 12862_2020_1720_MOESM1_ESM.png]

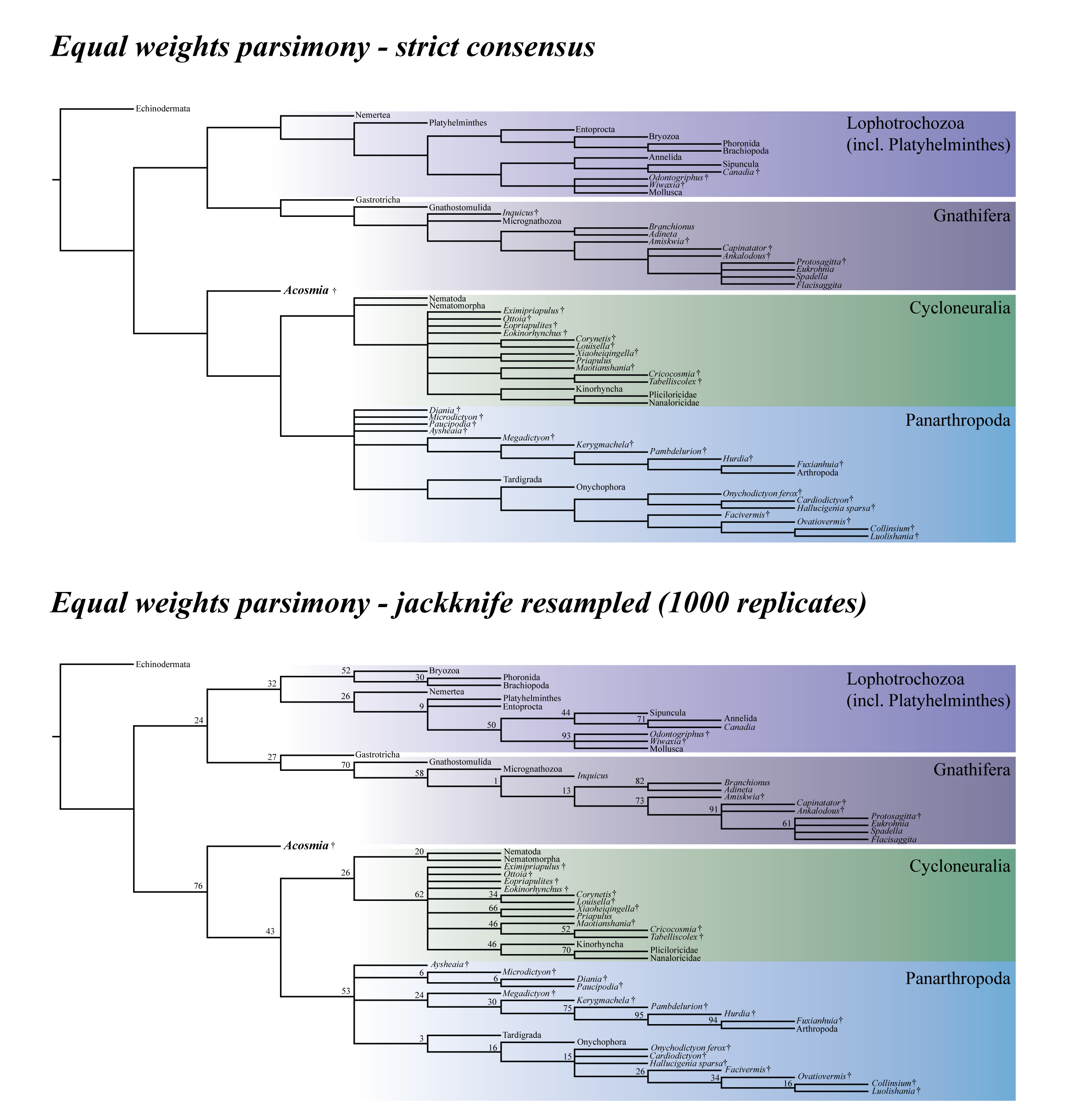

Supplement: Supplementary file 2 — Additional file 2: Fig. 2. Full results of equal weights parsimony-based tree searches. Daggers indicate fossil taxa. See section Methods – phylogenetic analyses for method details. [file 12862_2020_1720_MOESM2_ESM.png]

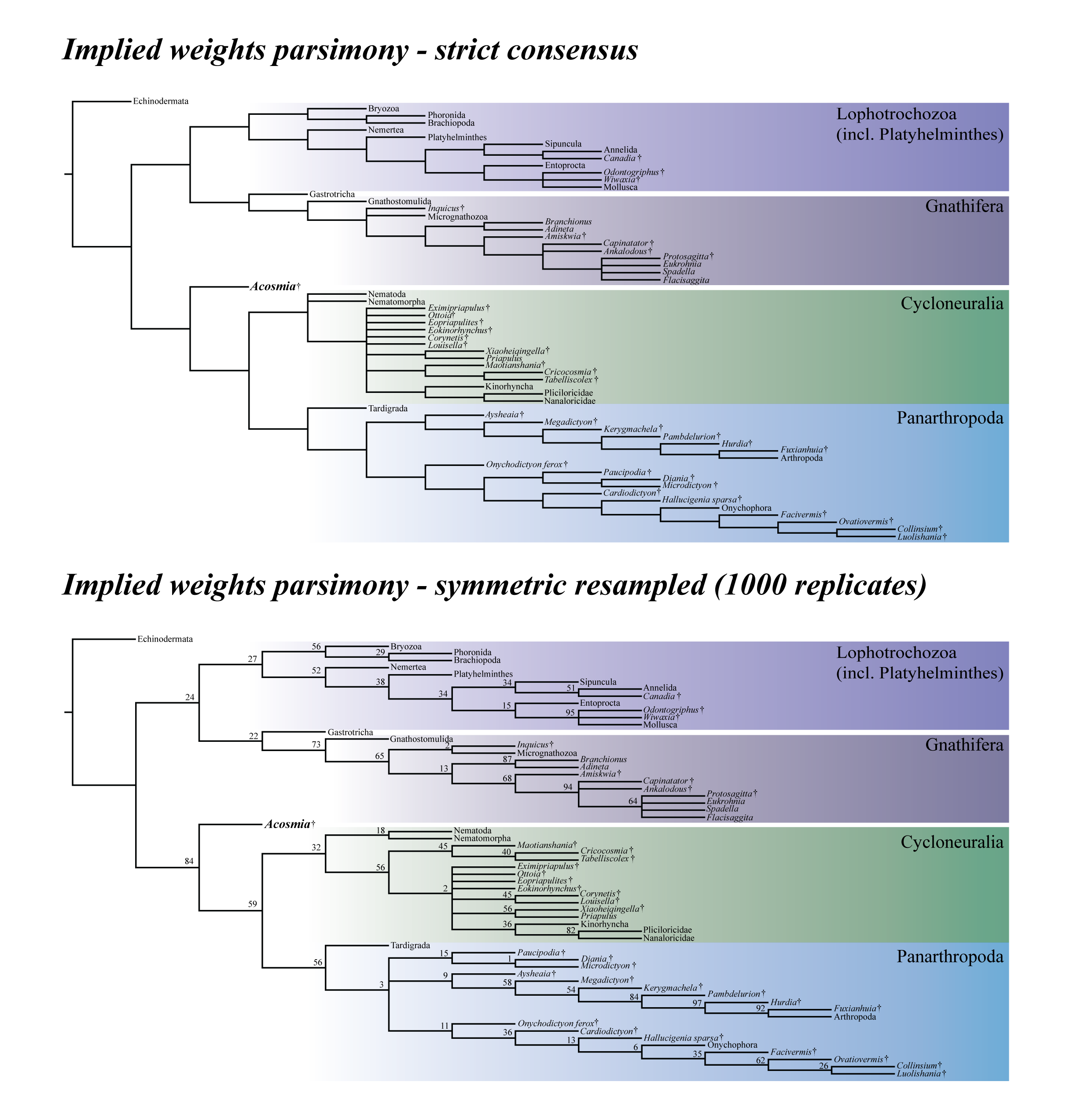

Supplement: Supplementary file 3 — Additional file 3: Fig. 3. Full results of implied weights (k=3) parsimony-based tree searches. Daggers indicate fossil taxa. See section Methods – phylogenetic analyses for method details. [file 12862_2020_1720_MOESM3_ESM.png]

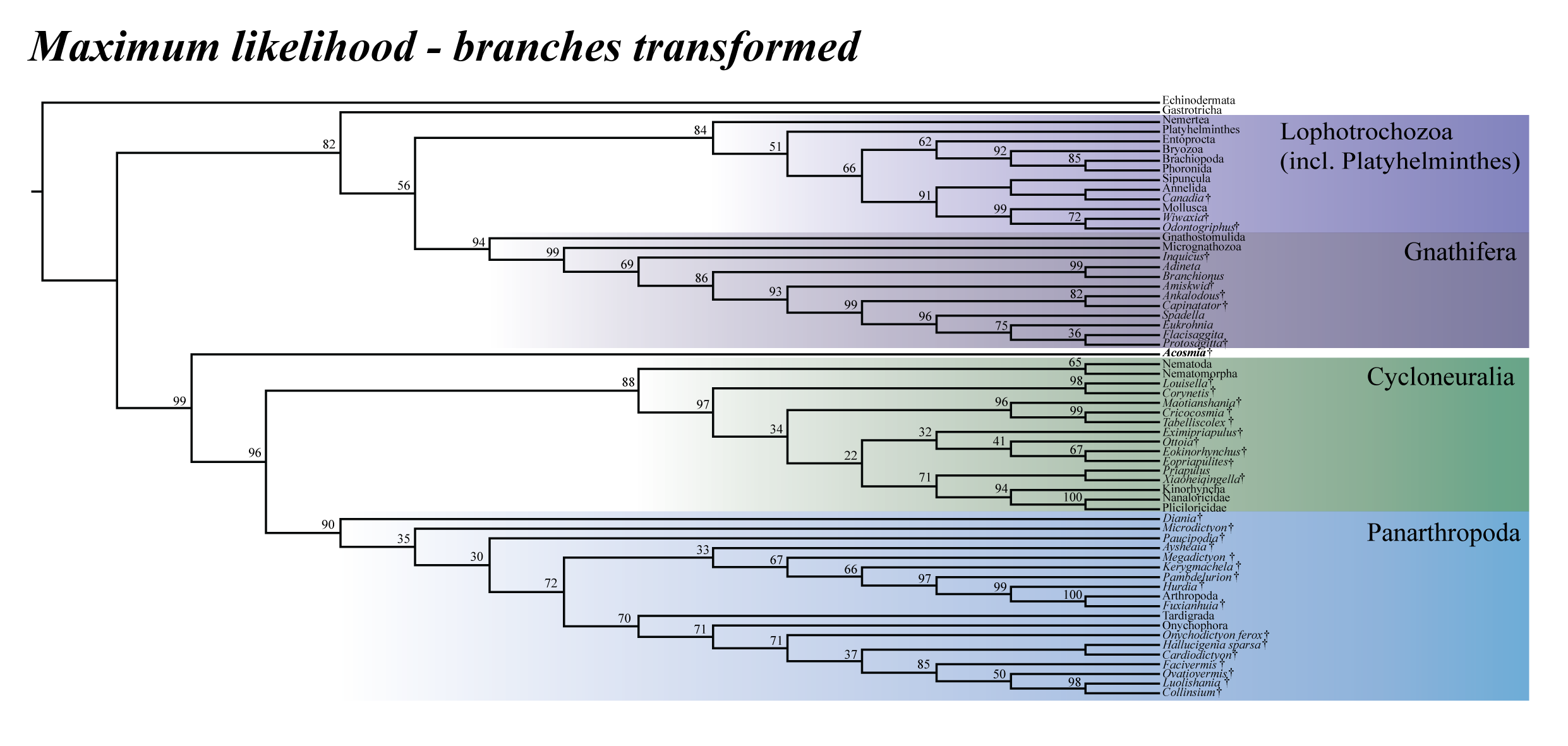

Supplement: Supplementary file 4 — Additional file 4: Fig. 4. Full topology of maximum likelihood tree search. Tree fully resolved and with branches transformed. Daggers indicate fossil taxa. See section Methods – phylogenetic analyses for method details. [file 12862_2020_1720_MOESM4_ESM.png]

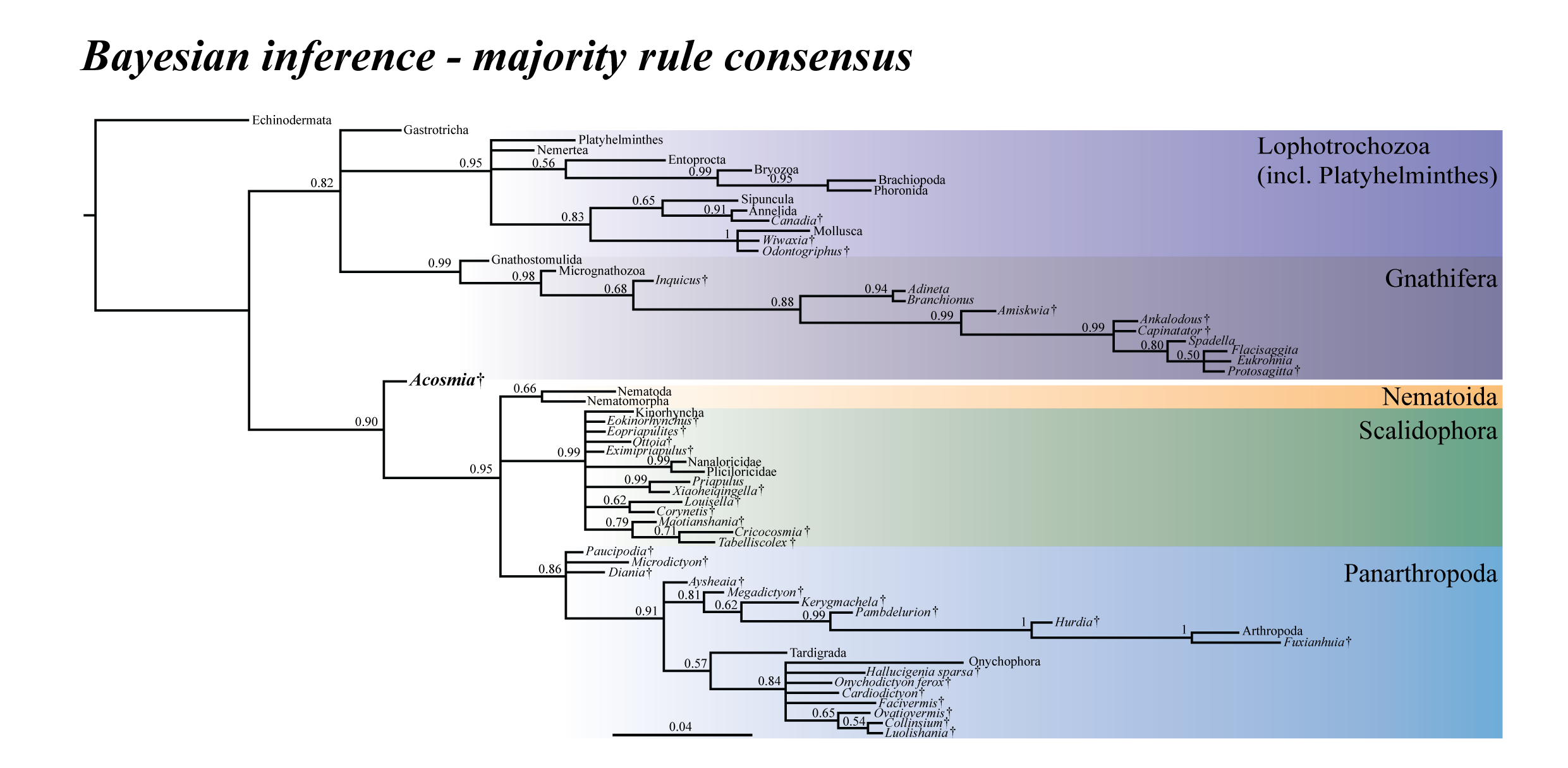

Supplement: Supplementary file 5 — Additional file 5: Fig. 5. Full topology of Bayesian inference tree search. Daggers indicate fossil taxa. See section Methods – phylogenetic analyses for method details. [file 12862_2020_1720_MOESM5_ESM.png]
