# Supplementary material for: Ancestral morphology of Ecdysozoa constrained by an early Cambrian stem group ecdysozoan
Source: BMC Evol Biol. 2020 Nov 23;20:156. doi: 10.1186/s12862-020-01720-6 (PMC7684930; doi:10.1186/s12862-020-01720-6)
Supplement: Supplementary file 6 — Additional file 6. List of morphological characters used in phylogenetic analyses. [file 12862_2020_1720_MOESM6_ESM.pdf]

Additional file 6. Morphological characters used in phylogenetic analyses. Characters 1-140 as in the dataset of Vinther & Parry (1), which includes characters from Ou et al. (2). Characters from those two studies are marked as such with a dagger (†) for Ou et al. and an asterisk for those from Vinther & Parry (*). Characters 141-185 are newly scored and references are provided. New characters are indicated as such.

1. †Collar complex

    0 absent

    1 present

2. †Multicellularity with extracellular matrix

    0 absent

    1 present

3. †Septate junctions (SJs)

    0 absent

    1 present

4. †Tight junctions (TJs)

    0 absent

    1 present

5. †Gap junctions (GJs)

    0 absent

    1 present

6. †Adherens junctions (AJs)

    0 absent

    1 present

7. †Hemidesmosomes

    0 absent

    1 present

8. †Epithelia

    0 absent

    1 present

9. †Basal laminae

    0 absent

    1 present

10. †Collagen

    0 absent

    1 present

11. †Nerve cells

0 absent

1 present

12. †Acetylcholine used as a neurotransmitter

0 absent

1 present

13. †Diffuse nervous system

0 absent

1 present

14. †Epidermis with pulsatite bodies

0 absent

1 present

15. *Ciliated epidermis.

0 absent

1 present

16. *Multiciliate epidermal cells

0 absent

1 present

17. *Ciliation restricted anteriorly.

0 absent

1 present

18. *Ciliated corona with paired nerves.

0 absent

1 present

19. *Ventral locomotory cilia.

0 absent

1 present

20. *Ventral surface modified into foot.

0 absent

1 present

21. †Xenacoelomorph cilia

0 absent

1 present

22. †Striated ciliary rootlets

0 absent

1 present

23. †Diploblasts built of two germ layers

0 absent

1 present

24. †Triploblasts built of three germ layers

0 absent

1 present

25. †Spiral cleavage

0 absent

1 present

26. *apical cross.

0 absent

1 present

27. †4d mesentoblast.

0 absent

1 present

28. †Colloblasts

0 absent

1 present

29. †Coelenteron (gastrovascular cavity)

0 absent

1 present

30. †Cnidae

0 absent

1 present

31. †Structure of mitochondrial DNA

0 circular

1 linear

32. †Actinopharynx

0 absent

1 present

33. †Siphonoglyph (sulcus)

0 absent

1 present

34. †Planulae

0 absent

1 present

35. †Polyp stage

0 absent

1 present

36. †Medusoid stage

0 absent

1 present

37. †Through-gut

0 absent

1 present

38. †U-shaped gut

0 absent

1 present

39. *Dorsal anus or anal pore.

0 absent

1 present

40. *Ventral mouth.

0 absent

1 present

41. Adult terminal mouth. Modified from Vinther and Parry (1) to clarify that this character describes the state of an anterior terminal mouth in the adult form (3–5).

0 absent

1 present

42. †Body divided with distinct inflated head and neck region.

0 absent

1 present

43. †Bipartite gut with cuticular pharynx.

0 absent

1 present

44. †Nephridia

0 absent

1 present

45. *Fate of blastopore.

0 protostomy

1 deuterostomy

2 amphistomy

3 unique

46. *annelid type cuticle

0 absent

1 present

47. †Body cuticle with chitin.

0 absent

1 present

48. †Body cuticle with alpha-chitin

0 absent

1 present

49. †Body cuticle molted

0 absent

1 present

50. †Lorica. Recoded as absent in Kinorhyncha.

0 absent

1 present

51. †lobopods or segmented limbs

0 absent

1 present

52. †limb type

0 lobopods

1 arthrodized limbs

53. *Anteriorly facing last pair of limbs.

0 absent

1 present

54. †Slime papillae

0 absent

1 present

55. †Telescoping mouth cone with protrudable stylets

0 absent

1 present

56. †Respiration via metameric tracheae and spiracles

0 absent

1 present

57. †Mixocoel (haemocoel) surrounded by segmented mesoderm

0 absent

1 present

58. †Teloblastic segmentation

0 absent

1 present

59. †Longitudinal ventral nerve cord(s)

0 absent

1 present

60. *Paired VNC. (Sipunculan form)

0 absent

1 present

61. *circumoral nerve ring.

0 absent

1 present

62. *Stomatogastric nerve plexi.

0 absent

1 present

63. Major ventral nerve plexus in trunk.

0 absent

1 present

64. Circum-pharyngeal, collar-shaped brain with anterior and posterior rings of perikarya separated by a ring-shaped neuropil.

0 absent

1 present

65. †Introvert with scalid rings

0 absent

1 present

66. †Flosculi

0 absent

1 present

67. †Immunoreactivity of horseradish peroxidase (HRP)

0 absent

1 present

68. †Trochophores

0 absent

1 present

69. *Prototroch.

0 absent

1 present

70. *Apical organ.

0 absent

1 present

71. *Apical organ with muscles extending to the hyposphere.

0 absent

1 present

72. †Segmental metanephridia sacculus

0 absent

1 present

73. †Chaetae.

0 absent

1 present

74. †Chaetae in bundles.

0 absent

1 present

75. †Mineralised chaetae.

0 absent

1 present

76. *Serially repeated chaetal bundles.

0 absent

1 present

77. †Parapodia with dorsal and ventral branches

0 absent

1 present

78. †Radula

0 absent

1 present

79. *Radula tooth rows.

0 few rows

1 many rows

80. *Chitinous pharyngeal structure.

0 absent

1 present

81. *Tube like support rods in jaw.

0 absent

1 present

82. *Grasping spines.

0 absent

1 present

83. *Teeth (as in Chaetognatha)

0 absent

1 present

84. *Anterior teeth (as in Chaetognatha).

0 absent

1 present

85. *Posterior teeth (as in Chaetognatha).

0 absent

1 present

86. *Jaw apparatus inside mastax.

0 absent

1 present

87. *Jaw apparatus forming a ventral membrane

0 absent

1 present

88. *Jaw forming clusters of homonomous elements.

0 absent

1 present

89. *Ctenidia.

0 absent

1 present

90. *Mantle cavity.

0 absent

1 present

91. †Eversible proboscis surrounded by rhynchocoel

0 absent

1 present

92. *Subterminal anus.

0 absent

1 present

93. *Tripartite body plan with septum.

0 absent

1 present

94. Posterior lateral trunk fins.

0 absent

1 present

95. Anterior lateral fins.

0 absent

1 present

96. *Caudal fin.

0 absent

1 present

97. *Fins supported by rays.

0 absent

1 present

98. *Phragms. .

0 absent

1 present

99. *Phragms in trunk.

0 absent

1 present

100. *Phragms in tail.

0 absent

1 present

101. *Stomatogastric nerve plexi.

0 absent

1 present

102. *Major nerve plexus in trunk.

0 absent

1 present

103. *Lateral sensory antennae.

0 absent

1 present

104. *Posterior adhesion structure.

0 absent

1 present

105. *Head tentacles.

0 absent

1 present

106. *Head with hood.

0 absent

1 present

107. *AP axis.

0 absent

1 present

108. *Dorsoventral axis

0 absent

1 present

109. †Origin of mesoderm

0 from the blastopore lips and as ectomesoderm

1 from the walls of the archenteron or neural crest

110. †Radial cleavage

0 absent

1 present

111. †coelom

0 absent

1 present

112. †Coelom formation

0 schizocoely

1 enterocoely

113. *Serially repeated paired coelomic cavities.

0 absent

1 present

114. †Trimeric coelom

0 absent

1 present

115. †Pharyngeal slits

0 absent

1 present

116. †Endostyle (or homologues)

0 absent

1 present

117. †Notochord

0 absent

1 present

118. †Stomochord

0 absent

1 present

119. †Haemal system with axial complex

0 absent

1 present

120. †Calcareous endoskeleton composed of separate ossicles

0 absent

1 present

121. †Tornaria type larva

0 absent

1 present

122. †Longitudinal dorsal nerve cord

0 absent

1 present

123. †Zig zag myomeres

0 absent

1 present

124. †Endothelium that lines the inner wall of blood vessels

0 absent

1 present

125. †Neural crest

0 absent

1 present

126. †Neurogenic placodes

0 absent

1 present

127. †Body symmetry

0 radial

1 bilateral

2 biradial

128. †Mesoglea

0 absent

1 present

129. †Cydippid larvae

0 absent

1 present

130. †Ciliary rosettes

0 absent

1 present

131. †Lophophore

0 absent

1 present

132. †Hox/ParaHox genes

0 absent

1 present

133. *ftz.

0 absent

1 present

134. *Ubx/abd-A.

0 absent

1 present

135. *AbdB.

0 absent

1 present

136. *lox5.

0 absent

1 present

137. *Duplication of UbdA into lox4 and lox2.

0 absent

1 present

138. *Post2.

0 absent

1 present

139. *Duplication into Post1 and Post2.

0 absent

1 present

140. *MedPost.

0 absent

1 present

Characters newly added to this matrix

141. Annulated cuticle. Character 37 in Smith & Caron [S6].

0 absent

1 present

142. Radial pharyngeal armature. Modified from character 13 in Smith & Caron [S6] (sclerotized pharyngeal 'teeth') to include all ecdysozoan radial pharyngeal armature (including kinorhynch pharyngeal styles, loriciferan placoids etc.).

0 absent

1 present

143. Posteriorly directed mouth opening. Modified from character 7 in Smith & Caron [S6].

0 absent

1 present

144. Serially repeated mid-gut glands. Character 53 in Smith & Caron [S6].

0 absent

1 present

145. Lobopodian-style ocelli. This character describes the paired and sessile visual units possessed by taxa such as *Hallucigenia sparsa* and *Luolishania longicruris* – see characters 16 – 18 in Smith & Caron [S6].

0 absent

1 present

146. Compound eyes. As in multiple visual units possessed by arthropods, again see characters 16 – 18 in Smith & Caron [S6].

0 absent

1 present

147. Cephalic sensillae **original character**. The anterior mechanosensroy and chemosensory organs in nematodes, forming rings in a 6 + 6 + 4 pattern around the anterior region [S7].

0 absent

1 present

148. Placids **original character**. The anterior plates that enclose the retracted introvert in kinorhynchs [S8].

0 absent

1 present

149. Elongate spines at the base of Zone II (collar). Character 13 in Wills et al. [S9] {Formatting Citation}.

0 absent

1 present

Inapplicable if taxon lacks scalid covered introvert

150. Introvert scalids arranged into parallel longitudinal rows. Modified from character 9 in Wills et al. [S9].

0 absent

1 present

Inapplicable if taxon lacks scalid covered introvert

151. Integument sclerotized and connected by arthrodial membranes. Character 35 in Smith & Caron [S6].

0 absent

1 present

152. Tergites associated with arthrodial membranes. Extension of Character 35 in Smith & Caron [S6].

0 absent

1 present

153. Sternites associated with arthrodial membranes. Character 36 in Smith & Caron [S6].

0 absent

1 present

154. Cephalic shield. Character 3 in Smith & Caron [S6].

0 absent

1 present

155. Musculature exclusively longitudinal **original character**. Nematoid bodyplans comprise only longitudinal muscles, with circular muscle completely absent [S8].

0 absent

1 present

156. Skeletal musculature metamerically arranged. Character 9 in Smith & Caron [S6].

0 absent

1 present

157. Jointed, reflexed introvert scalids **original character**. This character refers to the elongate anterior spinoscalids of loriciferans, which show a clear elbow-like joint [10].

0 absent

1 present

Inapplicable if taxon lacks scalid covered introvert

158. Eversible pharynx. Character 12 in Smith & Caron [S6].

0 absent

1 present

159. Paired metameric epidermal specialisations. Character 41 in Smith & Caron [S6].

0 absent

1 present

160. Metameric epidermal specialisations: spinose (as in hallucigeniids and luolishaniids)

0 absent

1 present

161. Metameric epidermal specialisations: net-like (as in Microdictyon)

0 absent

1 present

162. Metameric epidermal specialisations: saddle-like (as in Cardiodictyon)

0 absent

1 present

163. Metameric epidermal specialisations: node-like (as in Onychodictyon)

0 absent

1 present

164. Terminal claws on lobopods. Character 63 in Smith & Caron [S6].

0 absent

1 present

Inapplicable if taxon lacks paired appendages

165. Sclerites/claws consist of a stack of constituent elements (cone in cone structure). Character 48 in Smith & Caron [S6].

0 absent

1 present

Inapplicable if taxon lacks claws, sclerites etc.

166. Uniform appendages **original character**. This character describes the state of having metameric paired appendages lacking any differentiation (i.e. an absence of tagmosis). For example, the Chengjiang lobopodians *Paucipodia inermis, Diania cactiformis* and *Microdictyon sinicum*.

0 absent

1 present

Inapplicable if taxon lacks paired appendages

167. Pre-ocular appendage pair structurally differentiated from trunk appendages. Character 20 in Smith & Caron [S6].

0 absent

1 present

Inapplicable if taxon lacks paired appendages

168. Pre-ocular appendage pair antenniform **original character**. This character describes the antenniform anteriormost appendages (inferred to be protocerebral) of some onychophorans and some arthropods.

0 absent

1 present

Inapplicable if taxon lacks paired appendages

169. Spines/spinules on pre-ocular (protocerebral) appendage. Character 30 in Smith & Caron [S6].

0 absent

1 present

Inapplicable if taxon lacks paired appendages

170. Sclerotization of pre-ocular (protocerebral) limb pair. Character 21 in Smith & Caron [S6].

0 absent

1 present

Inapplicable if taxon lacks paired appendages

171. Pre-ocular (protocerebral) limb pair with arthrodial membranes. Character 22 in Smith & Caron [S6].

0 absent

1 present

Inapplicable if taxon lacks paired appendages

172. Pre-ocular (protocerebral) appendages mechanically fused. Character 28 in Smith & Caron [S6].

0 absent

1 present

Inapplicable if taxon lacks paired appendages

173. Deutocerebral limb pair structurally differentiated from trunk appendages. Character 24 in Smith & Caron [S6].

0 absent

1 present

Inapplicable if taxon lacks paired appendages

174. Sclerotized post-ocular (post-protocerebral) body appendages with arthrodial membranes. Character 19 in Smith & Caron [S6].

0 absent

1 present

Inapplicable if taxon lacks paired appendages

175. Laterally expanded swimming flap as inner branch of lobopod. Character 23 in Smith & Caron [S6].

0 absent

1 present

Inapplicable if taxon lacks paired appendages

176. Biramy (exite and endopod fused). Character 57 in Smith & Caron [S6].

0 absent

1 present

Inapplicable if taxon lacks paired appendages

177. Anterior-posterior tagmosis of non-arthropodized appendages **original character**. Whereas some lobopodians have appendages of uniform construction (e.g. *Paucipodia inermis*), and some have a single specialised anterior appendage (e.g. *Aysheaia pedunculata*), some lobopodians have multiple specialised anterior appendages – resulting in an anterior-posterior tagmosis (e.g. *Hallucigenia sparsa*, *Cardiodictyon catenulum*, Luolishaniidae and Onychophora).

0 absent

1 present

Inapplicable if taxon lacks paired appendages

178. Elongated anterior lobopodous limbs, differentiated from shorter posterior two short limbs. Character 72 in Yang et al. [S10].

0 absent

1 present

Inapplicable if taxon lacks paired appendages

179. Setae on anterior specialized lobopods. Character 60 in Smith & Caron [S6].

0 absent

1 present

Inapplicable if taxon lacks paired appendages

180. Caudal appendage. Character 45 in Wills et al. [S9].

0 absent

1 present

181. Two rows of ventral trunk papillae. Character 30 in Wills et al. [S9].

0 absent

1 present

182. Posterior hooks in a ring. Character 30 in Wills et al. [S9].

0 absent

1 present

183. Posterior pair of hooks. Character 30 in Wills et al. [S9].

0 absent

1 present

184. Swollen, hook-bearing posterior **original character***. This character describes the anchoring mass of *Facivermis yunnanicus* [S11]

0 absent

1 present

185. Radially arranged circumoral structures. Character 9 in Smith & Caron [S6].

0 absent

1 present

Supplementary references

1. Vinther J, Parry LA. Bilateral Jaw Elements in Amiskwia sagittiformis Bridge the Morphological Gap between Gnathiferans and Chaetognaths. Curr Biol. 2019;29(5):881-888

2. Ou Q, Han J, Zhang ZF, Shu DG, Sun G, Mayer G. Three Cambrian fossils assembled into an extinct body plan of cnidarian affinity. Proc Natl Acad Sci. 2017;114(33):8835–8840.

3. Ortega-Hernández J, Janssen R, Budd GE. Origin and evolution of the panarthropod head – A palaeobiological and developmental perspective. Arthropod Struct Dev. 2017;46(3):354–79.

4. Ortega-Hernández J, Janssen R, Budd GE. The last common ancestor of Ecdysozoa had an adult terminal mouth. Arthropod Struct Dev. 2019;49:155–8.

5. Nielsen C. Was the ancestral panarthropod mouth ventral or terminal? Arthropod Struct Dev. 2019;49:152–4.

6. Smith MR, Caron JB. *Hallucigenia's* head and the pharyngeal armature of early ecdysozoans. Nature. 2015;523(7558):75–8.

7. Lee DL. The Biology of Nematodes. London: Taylor & Francis; 2002.

8. Schmidt-Rhaesa A. Handbook of Zoology Gastrotricha, Cycloneuralia and Gnathifera Volume 1: Nematomorpha, Priapulida, Kinorhyncha, Loricifera. Berlin: Walter de Gruyter GmbH; 2013.

9. Wills MA, Gerber S, Ruta M, Hughes M. The disparity of priapulid, archaeopriapulid and palaeoscolecid worms in the light of new data. 2012;25:2056–76.

10. Yang J, Ortega-Hernández J, Gerber S, Butterfield NJ, Hou J, Lan T, Zhang XG. A superarmored lobopodian from the Cambrian of China and early disparity in the evolution of Onychophora. Proc Natl Acad Sci. 2015;112(28):8678–83

11. Howard RJ, Hou XG, Edgecombe GE, Salge T, Shi XM, Ma XY. A Tube-Dwelling Early Cambrian Lobopodian. Curr Biol. 2020;30:1-8.
